# Metagenomic Analysis Revealed Methylamine and Ureide Utilization of Soybean-Associated *Methylobacterium*

**DOI:** 10.1264/jsme2.ME16035

**Published:** 2016-07-15

**Authors:** Tomoyuki Minami, Misue Anda, Hisayuki Mitsui, Masayuki Sugawara, Takakazu Kaneko, Shusei Sato, Seishi Ikeda, Takashi Okubo, Hirohito Tsurumaru, Kiwamu Minamisawa

**Affiliations:** 1 Graduate School of Life Sciences, Tohoku University2–1–1 Katahira, Aoba-ku, Sendai 980–85577Japan; 2 Kazusa DNA Research Institute2–6–7 Kazusa-kamatari, Kisarazu, Chiba 292–0818Japan

**Keywords:** *Methylobacterium*, soybean, metagenome, methylamine, ureide

## Abstract

*Methylobacterium* inhabits the phyllosphere of a large number of plants. We herein report the results of comparative metagenome analyses on methylobacterial communities of soybean plants grown in an experimental field in Tohoku University (Kashimadai, Miyagi, Japan). *Methylobacterium* was identified as the most dominant genus (33%) among bacteria inhabiting soybean stems. We classified plant-derived *Methylobacterium* species into Groups I, II, and III based on 16S rRNA gene sequences, and found that Group I members (phylogenetically close to *M. extorquens*) were dominant in soybean-associated *Methylobacterium*. By comparing 29 genomes, we found that all Group I members possessed a complete set of genes for the *N*-methylglutamate pathway for methylamine utilization, and genes for urea degradation (urea carboxylase, urea amidolyase, and conventional urease). Only Group I members and soybean methylobacterial isolates grew in a culture supplemented with methylamine as the sole carbon source. They utilized urea or allantoin (a urea-related compound in legumes) as the sole nitrogen source; however, group III also utilized these compounds. The utilization of allantoin may be crucial in soybean-bacterial interactions because allantoin is a transported form of fixed nitrogen in legume plants. Soybean-derived Group I strain AMS5 colonized the model legume *Lotus japonicus* well. A comparison among the 29 genomes of plant-derived and other strains suggested that several candidate genes are involved in plant colonization such as *csgG* (curli fimbriae). Genes for the *N*-methylglutamate pathway and curli fimbriae were more abundant in soybean microbiomes than in rice microbiomes in the field. Based on these results, we discuss the lifestyle of *Methylobacterium* in the legume phyllosphere.

*Methylobacterium* is a genus of facultative methylotrophic bacteria that utilize not only multicarbon compounds (22), but also C1 compounds such as methanol (16, 19). *Methylobacterium* has been isolated from various environments including water (21), pink-pigmented biofilms (70, 71), soils (42), and plant tissues (2, 35). Culture-dependent (2, 33) and culture-independent analyses (13, 37) have revealed that *Methylobacterium* resides ubiquitously in the plants of various species such as soybean (2, 27), rice (30, 37, 49), *Arabidopsis* (*Arabidopsis thaliana*) (13, 35), and poplar (1).

Plant-associated *Methylobacterium* has been suggested to play beneficial roles in plant growth presumably via changes in the hormone balance by auxin and cytokinin (46) and by aminocyclopropane-1-carboxylate (ACC) deaminase (41). Phenylalanine ammonia-lyase, β-1,3-glucanase, and peroxidase were found to be activated in plants inoculated with *Methylobacterium* (40); these enzymes are associated with induced systemic resistance to pathogens (38).

*Methylobacterium* has been suggested to utilize methanol emitted from plant stoma (46) as a by-product of plant pectin metabolism (18). Under competitive conditions, wild-type *Methylobacterium extorquens* AM1 colonizes the model legume, *Medicago truncatula*, better than two mutants deficient in the methanol dehydrogenase (*mxaFI*) or tetrahydromethanopterin biosynthesis (*mptG*) gene (60). However, the abundance of the two mutants was almost the same as that of wild-type AM1 when each strain was inoculated individually (60). Thus, methanol utilization is advantageous, but not essential for plant colonization by *Methylobacterium* (60). Bacterial propagation expands the colonized area during the rapid growth of plant shoots (67); this propagation requires a sufficient supply of C and N substrates from host plants to bacteria. Therefore, *Methylobacterium* may utilize substrates other than methanol for efficient plant colonization. N fertilization and nodulation have been shown to frequently affect the abundance of *Methylobacterium* in the shoots of field-grown soybean plants (25, 27, 28, 48).

Although a large number of *Methylobacterium* genomes (39) and metagenomic data for plant microbiomes (13) have been published, the carbon and nitrogen sources (other than methanol) that allow *Methylobacterium* to propagate in the phyllosphere are poorly understood. Therefore, we compared *Methylobacterium* genomes in combination with a phylogenetic analysis to identify the carbon and nitrogen sources for these bacteria. A specific phylogenetic group grew using methylamine as the sole carbon source, while all methylobacteria tested utilized ureide and urea as the sole nitrogen source. We also tested the abundance of genes relevant to these processes among the metagenomic data of bacterial microbiomes associated with soybean and rice (49) grown in the same field (36). Genes for methylamine utilization and *csgG* (encoding curli fimbriae) were more abundant in soybean microbiomes than in rice microbiomes in the field.

## Materials and Methods

### Metagenome analysis

Soybean plants (*Glycine max.* [L.] Merr. cv. Enrei) were grown as described previously (28). Two sets of soybean stem samples (termed SoyJp1 and SoyJp2 in the present study) were two biological replicates. Each replicate was derived from four soybean plants that were grown under identical field conditions. Bacterial cells were isolated from the stems by density gradient centrifugation (26, 29). A DNase treatment was added to the procedure in order to remove plant DNA (29). After the final bacterial cell suspension was incubated in the presence of recombinant DNase I (Takara, Otsu, Japan) at 37°C for 20 min, the reaction was terminated by the addition of 0.5 M EDTA at a final concentration of 25 mM. Total DNA in the enriched bacterial cells was extracted using the bead-beating method of Ikeda *et al.* (26, 27).

Total bacterial DNA was sequenced using a 454 GS FLX+ genome sequencer (Roche Diagnostics, Tokyo, Japan). Low-quality and duplicated sequences were removed by a 454 Replicate Filter, and the remaining reads were grouped on the basis of phylogeny and a functional analysis using the metagenomics RAST server (43). Phylogenetic assignment was conducted in the best-hit classification mode using the M5NR and M5RNA databases with a cut-off e-value of 10^−10^. Functional assignment was conducted in the “all annotations” mode using the GenBank database with a cut-off e-value of 10^−10^. The relative abundance of a particular methylobacterial gene in the metagenome was assessed on the basis of the number of read hits in a TBLASTX search using CLC Genomics Workbench (CLC Bio, Arhus, Denmark). The hit number was found to cover reads with e-values <10^−10^ and amino acid sequence identities for which there were no hits in the BLASTP search of the non-redundant protein sequence database at the NCBI. Hit numbers were normalized to gene lengths.

### Phylogenetic analysis

16S rRNA sequence data were obtained from the GenBank database. Phylogenetic trees were constructed with the Clustal W program (64) using the neighbor-joining method (57) and tree topology was evaluated with 1,000 bootstrap trials using MEGA version 6.0 (63).

### Comparison of genome sequences

Genes were clustered according to the amino acid sequence identities of the encoded proteins (≥70%) using the complete genome sequences of *Methylobacterium* species and CD-HIT (24) with default parameters, except that aL was set at 0.7, aS at 0.7, and s at 0.9. The genes identified were then used as queries for BLASTP searches on *Methylobacterium* strain sequences, and hits with identities ≥30% and e-values <10^−10^ were regarded as having significant similarity. Comparisons of *Methylobacterium* genes were performed by GenomeMatcher (47).

### Enumeration and phylogenetic identification of methylobacteria from soybean plants

The leaves and stems of soybean plants grown for 91 d were macerated in sterilized water with a mortar and pestle, serially diluted with sterilized water, and plated on AMS agar plates (68) containing 10 μg mL^−1^ cycloheximide and a carbon source (20 mM methanol or methylamine hydrochloride). After being incubated at 28°C for 6 d, pink colonies (considered to be methylobacteria) were counted and randomly selected for single colony isolation on AMS agar containing 20 mM methanol. After the first colonies that appeared had been restreaked on the same medium, bacterial colonies were selected again to ensure purity. Template DNA was prepared as described (58), partial 16S rRNA genes were sequenced (2) and a phylogenetic tree was constructed (57).

### Growth tests of methylobacteria from soybean and rice plants

In order to test different carbon sources for growth, cell suspensions (5 μL) were spotted on AMS agar plates (68) containing 123 mM methanol or 123 mM methylamine hydrochloride and incubated at 28°C for 5 d. The same cell suspensions were spotted on methanol-containing AMS agar plates in which NiCl_2_ was omitted and NH_4_Cl was replaced with 4.7 mM urea or 2.3 mM allantoin to test different nitrogen sources.

### Plant inoculation test

The strains and plasmids used for plant inoculation tests are shown in Table S1. *Methylobacterium* sp. AMS5 and *M. extorquens* AM1 were tagged with β-glucuronidase (to obtain the AMS5g and AM1g strains, respectively) using mTn5SS*gus*A20 (69), which was conjugated from *Escherichia coli* DH5α through triparental mating with *E. coli* MT616 as a helper strain (20) and selection for resistance to streptomycin, spectinomycin, and polymyxin B. These strains were grown at 30°C in AMS medium. The seeds of *Lotus japonicus* cv. Gifu B-129 were scuffed with sandpaper, sterilized with 70% (v/v) ethanol and 0.8% (w/v) sodium hypochlorite solution, rinsed, and immersed in sterilized water for 4 h and then in a suspension of AMS5g or AM1g cells (2×10^7^ cells mL^−1^) for 4 h. Seeds were sown in sterilized vermiculite soaked in B&D medium (8) in Leonard’s jars, which were placed in a growth chamber under a 16-h light and 8-h dark cycle at 22°C. Ten d later, *Mesorhizobium loti* MAFF303099 cells, which had grown in TY medium (58) at 30°C, were added to vermiculite at 2.5×10^7^ cells per plant. A histochemical assay of methylobacteria was performed 10, 23, and 40 d after sowing as described previously (66) with slight modifications. The plants were immersed in ice-cold acetone for 15 min and then transferred into GUS staining solution (0.5 mM potassium ferricyanide and ferrocyanide, 0.1 mg mL^−1^ 5-bromo-4-chloro-3-indolyl-β-d-glucuronide) with vacuum infiltration for 30 min and an overnight incubation at 25°C. Plants were rinsed with 70% ethanol and clearing solution (acetic acid:ethanol = 1:6 [v/v]) and observed under a SZX12 stereo-microscope (Olympus, Tokyo, Japan).

### Accession number

The metagenomic DNA sequences analyzed in the present study were deposited in the MG-RAST (Project ID mgp18755; Names 4706424.3 [SoyJp1] and 4706425.3 [SoyJp2]) and DDBJ Sequence Read Archive (accession no. DRA004474). The nucleotide sequences of the 16S rRNA genes of 24 soybean isolates of *Methylobacterium* were deposited in DDBJ (LC127483–LC127506).

## Results and Discussion

### Statistical summary of metagenomic data

Two sets (SoyJp1 and SoyJp2) of metagenome sequences were examined from the bacterial communities of the stems of field-grown soybean plants in an experimental field in Tohoku University (Kashimadai, Miyagi, Japan) for which metagenomic data from rice plants (RiceJp1 and RiceJp2) are available (49). A statistical summary of the metagenomic data is shown in Table S2. In both soybean sets, *Alphaproteobacteria* showed the highest relative abundance (61.44–61.56%) at the phylum level (Table S3), and the most dominant genus was *Methylobacterium* (33.33–33.62%), followed by *Agrobacterium* and *Rhizobium* (Table S3, Fig. S1). In rice, the order of the major genera was similar, although the abundance of *Methylobacterium* in both sets was lower (7.91–13.5%; Table S3, Fig. S1).

### Grouping of plant-associated methylobacteria

In the phylogenetic analysis, we collected 16S rRNA gene sequences from the complete genomes of 11 strains and draft genomes of 23 strains (Table S4). The general features of the complete genomes are shown in Table S5. We also included the 16S rRNA gene sequences of the representative strains of 8 operational taxonomic units (OTUs M1–M8), which were all isolated from soybean plants (2). Minami *et al.* (44) reported the complete genome sequence of a representative strain AMS5 in a major OTU M4 (2). AMS5 was included in our phylogenetic analysis. Type strains of *Methylobacterium* species were added to the phylogenetic analysis based on the 16S rRNA gene regardless of the presence or absence of their genome information.

**Fig. 1:**
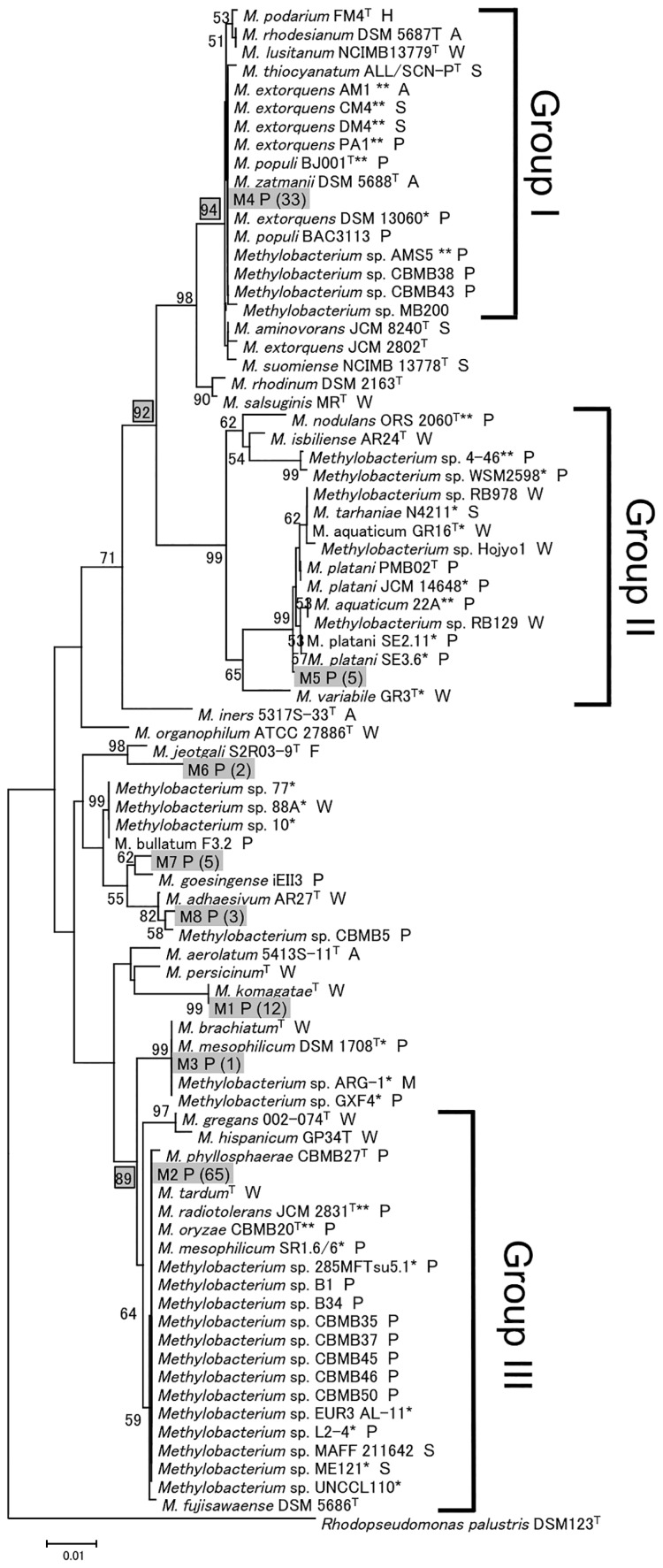
Neighbor-joining phylogenetic tree of 16S rRNA genes from *Methylobacterium* species. The scale bar represents the substitution number per site. The numbers at nodes are bootstrap values (%; values <50 are not shown). M1 to M8 are the OTUs described previously (2); the numbers in parentheses after each OTU name are the numbers of isolates assigned to the OTU. Strain sources are indicated after the strain names: A, air; F, food; H, human; P, plant; S, soil; W, water. *draft genome sequence published; **complete genome sequence published.

The phylogenetic tree with isolation sources is shown in Fig. 1. Plant-derived isolates (“P” in Fig. 1) formed three groups (I–III). Group I (bootstrap value, 94%; Fig. 1) contained all strains of plant-derived *M. extorquens* and strains from soybean (AMS5), *A. thaliana* (PA1), and poplar (BJ001). Group II (bootstrap value, 92%) contained *M. nodulans* ORS2060 (32), *Methylobacterium* sp. 4–46, and *Methylobacterium* sp. WSM2598, which are able to nodulate legumes (*Crotalaria podocarpa* [ORS2060] or *Lotononis bainesii* [4–46 and WSM2598]) for symbiotic nitrogen fixation (3, 32). Group III (bootstrap value, 89%) included *M. radiotolerans* JCM2831, *M. oryzae* CBMB20 from rice plants, *M. mesophilicum* SR1.67 from orange, and *Methylobacterium* sp. L2–4 from *Jatropha curcas*. Several strains derived from plants were located between Groups II and III; the bootstrap values (<50%) were too low to assign them to a group (Fig. 1) and were referred to as “others” in the present study. All 11 strains with complete genomes were classified into Group I (strains AM1, CM4, DM4, PA1, BJ001, and AMS5), Group II (ORS2060, 4–46, and 22A), or Group III (JCM2831 and CBMB20) (double asterisks in Fig. 1).

### Comparison of *Methylobacterium* groups in soybean and rice microbiomes

**Fig. 2:**
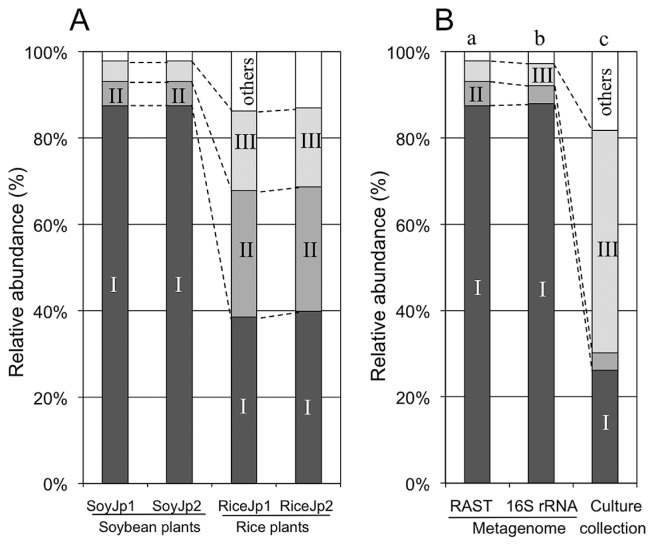
Relative abundance of Groups I–III in different *Methylobacterium* populations revealed by a metagenome analysis. (A) Soybean metagenomes (SoyJp1 and SoyJp2) vs. rice metagenomes (RiceJp1 and RiceJp1). Metagenomes were obtained from plants grown at the same site. (B) The SoyJp1 dataset vs. a collection of *Methylobacterium* isolates. The SoyJp1 dataset was analyzed using the MG-RAST server (all sequence reads) (a), and on the basis of 16S rRNA (1,324 sequence reads) (b). The culture collection contained 126 isolates from soybean plants grown at the same site (c) (2).

The relative abundance of Group I methylobacteria was much higher than that of Groups II and II in soybean microbiomes (Fig. 2A). However, the relative abundance of Group I was lower in rice microbiomes than in soybean microbiomes (Fig. 2A). Thus, our culture-independent study suggests that Group I members of *Methylobacterium* are dominant in soybean microbiomes. When previous culture-dependent findings (2) was compared with the overall diversity of *Methylobacterium*, the relative abundance of Group I (Fig. 2B) measured in a culture-dependent study (2) was markedly lower than that in metagenome (Fig. 2B) and 16S rRNA gene analyses (Fig. 2B). These results suggest the presence of a typical culture bias in the methylobacterial community composition.

### Group-specific genes in methylobacterial genomes

**Fig. 3:**
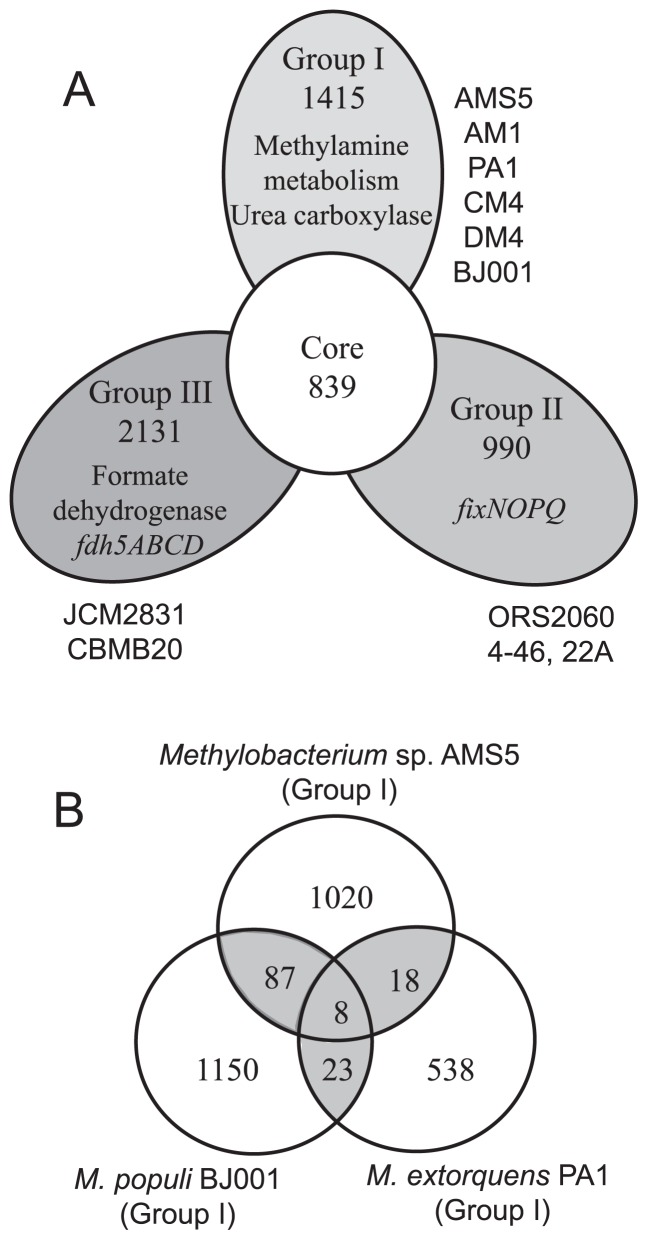
Distribution of orthologous genes within the genus *Methylobacterium*. (A) Flower plot: the numbers of genes shared by all strains of the same group or all strains in all three groups (center). (B) Venn diagram: genes shared by three plant-associated Group I strains; genes shared with other Group I members were excluded.

Using the 11 complete genomes in the CD-HIT analysis, we detected unique genes and those shared among the three groups (Table S5). We found 1,415 unique genes in Group I, 990 in Group II, and 2,131 in Group III (Fig. 3A). We searched for group-specific genes relevant to carbon/nitrogen metabolism and respiration and found *mgdD* (*N*-methylglutamate dehydrogenase gene) and the urea carboxylase gene (Group I), *fixNOPQ* (Group II), and *fdh5* (Group III) (Table 1). We first analyzed Group I-specific genes in detail.

### Group I-specific genes for C1 compound metabolism

**Fig. 4:**
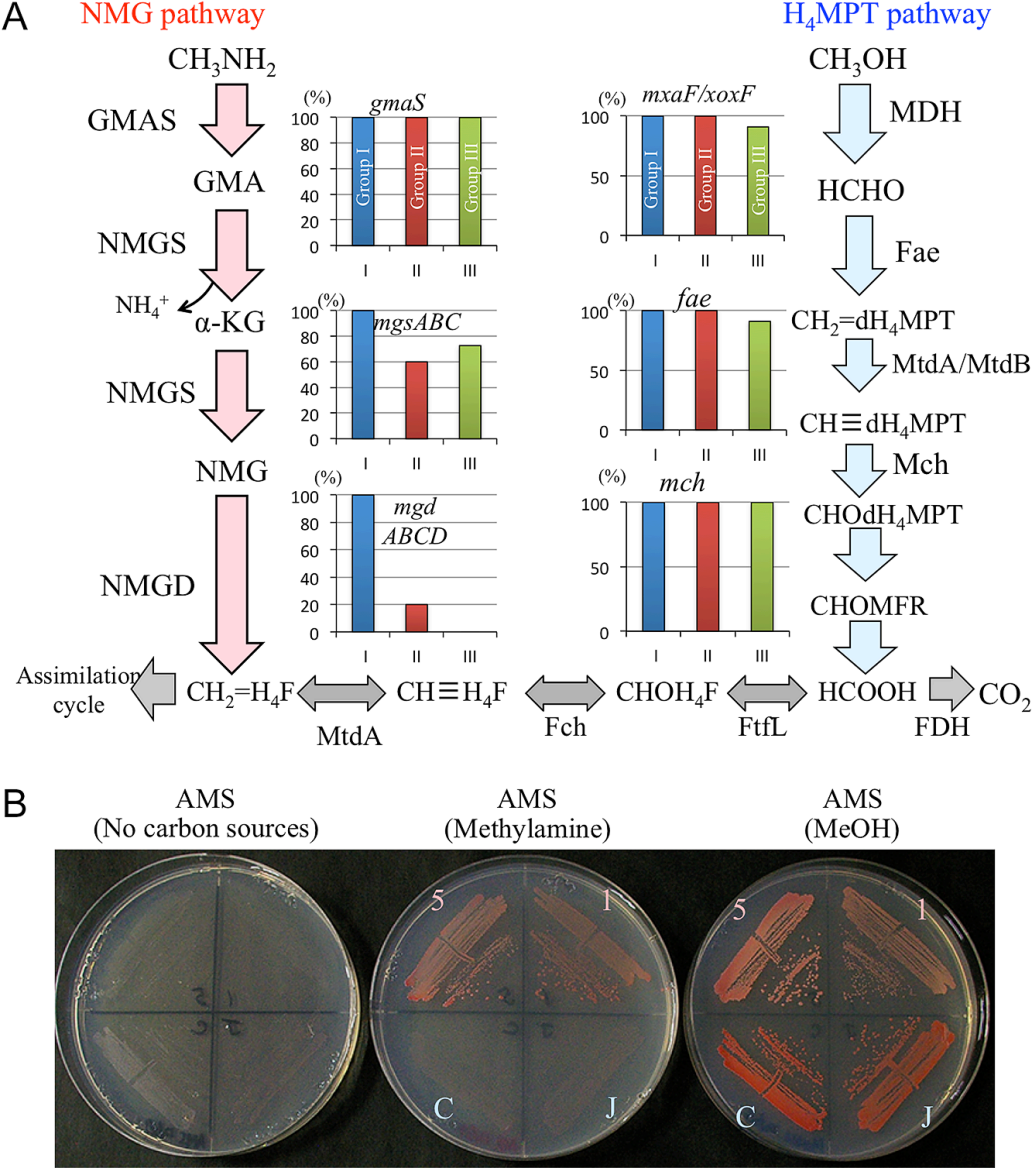
Distribution of genes involved in methylamine and methanol metabolism among *Methylobacterium* species and the ability of *Methylobacterium* strains to use methylamine and methanol as sole carbon sources. (A) Percentage of strains in each group that have genes responsible for each step; pathway maps are shown next to the graphs. Abbreviations for enzymes: Fae, formaldehyde-activating enzyme; Fch, methenyl-H_4_F cyclohydrolase; FDH, formate dehydrogenase; Fhc, formyltransferase/hydrolase complex; FtfL, formate-tetrahydrofolate ligase; Mch, methenyl-dH_4_MPT cyclohydrolase; MDH, methanol dehydrogenase; MtdA and MtdB, methylene-tetrahydromethanopterin dehydrogenase. Abbreviations for compounds: CH_2_=dH_4_MPT, methylene-dH_4_MPT; CH≡dH_4_MPT, methenyl-dephospho H_4_MPT; CHOdH_4_MPT, formyl dH_4_MPT; MFR, methanofuran; CHOMFR, formyl-MFR; H_4_F, tetrahydrofolate; CHOH_4_F, formyl-H_4_F; CH≡H_4_F, methenyl H_4_F; dH_4_MPT, dephosphotetrahydromethanopterin; GMA, γ-glutamylmethylamide; NMG, *N*-methylglutamate; α-KG, α-ketoglutarate. (B) Growth test without a carbon source or with methylamine or methanol as the sole carbon source for 7 d. 5, *Methylobacterium* sp. AMS5 (Group I); 1, *M. extorquens* AM1 (Group I); C, *M. oryzae* CBMB20 (Group III); J, *M. radiotolerans* JCM2831 (Group III).

The *mgdD* gene is a part of the *mgdABCD* operon encoding *N*-methylglutamate dehydrogenase (NMGD) (23). NMGD is involved in the *N*-methylglutamate (NMG) pathway in *Methylobacterium* methylamine metabolism (23). Gruffaz *et al.* (23) recently found that the NMG pathway rather than canonical methylamine dehydrogenase (MaDH) encoded by the *mauAB* genes (9) prevails in *Methylobacterium* (Fig. S2). The outlines of the NMG pathway for methylamine oxidation and the tetrahydromethanopterin (H_4_MPT) pathway for methanol oxidation are shown in Fig. 4A, and a detailed pathway map is presented in Fig. S2. The NMG pathway is mediated by *gmaS* encoding γ-glutamylmethylamide (GMA) synthetase, *mgsABC* encoding NMG synthase (NMGS), and *mgdABCD* (Fig. 4A). Note that NMGS mediates two steps, from GMA to α-ketoglutarate (α-KG) and from α-KG to NMG (Fig. 4A).

The distribution of the genes for the NMG and H_4_MPT pathways was searched among 11 complete and 18 draft genomes (Fig. 4, Table S4). All strains possessed the *gmaS* gene. All Group I strains and most strains in Groups II and III had *mgsABC*. However, 80% of Group II strains and all Group III strains lacked the *mgdABCD* genes encoding NMGD, which catalyzes the last step in the NMG pathway, whereas all Group I strains possessed *mgdABCD*. These results strongly suggest that the NMG pathway is complete mainly within Group I, which is supported by strong synteny in the gene cluster for the NMG pathway (*gmsS/mgsABC/mgdABCD*) (Fig. S3). In contrast, genes for the H_4_MPT pathway were conserved well in Groups I, II, and III (Fig. 4A), suggesting that all strains are able to oxidize methanol. In addition, a few strains of Group II and III lacked the *mxaF* gene, whereas all strains had the *xoxF* gene (data not shown).

We then grew the strains of Groups I and III on AMS agar media containing methylamine or methanol as the sole carbon sources. In the presence of methylamine, Group III strains (*M. radiotolerans* JCM2831 and *M. oryzae* CBMB20) did not grow, whereas Group I strains (*M. extorquens* AM1 and *Methylobacterium* sp. AMS5) did (Fig. 4B). All four strains grew well in the presence of MeOH (Fig. 4B). This result may be explained by the lack of complete sets of genes for the NMG pathway in Group III (Fig. 4A).

### NMG pathway genes in soybean and rice microbiomes

**Fig. 5:**
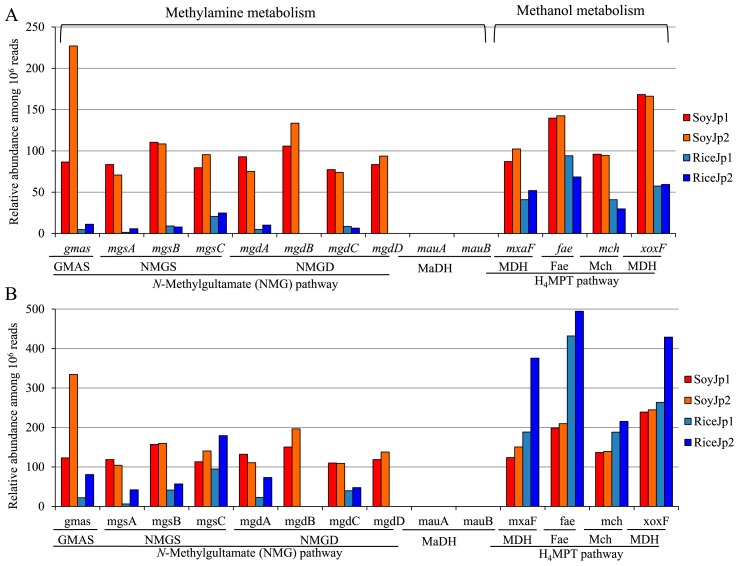
Relative abundance of genes involved in methanol and methylamine oxidation. The relative abundance of genes was normalized to the length of each gene. (A) Total reads that were divided by the number of metagenome reads. (B) Total reads that were divided by the proportion of methylobacteria in each of the four sets. Fae, formaldehyde-activating enzyme; GMAS, gamma-glutamylmethylamide synthetase; NMGD, N-methyl glutamate dehydrogenase; NMGS, N-methyl glutamate synthase; MaDH, methylamine dehydrogenase; MDH, methanol dehydrogenase; Mch, methenyl-dH_4_MPT cyclohydrolase; H_4_MPT, tetrahydromethanopterin.

The abundance of genes for the NMG and H_4_MPT pathways and for methylamine dehydrogenase (MaDH, a canonical methylamine metabolism enzyme) in the metagenomes of soybean and rice microbiomes is shown in Fig. 5A. The abundance of NMG pathway genes in both soybean sets (71 to 227 per 10^6^ reads) was higher than that in both rice data sets (0 to 24.8 per 10^6^ reads). The *mgdBD* genes were not detected in either rice set. We did not detect the genes encoding MaDH in any metagenome data sets (Fig. 5), suggesting that the NMG pathway is responsible for methylamine metabolism. Genes for the H_4_MPT pathway were abundant in all metagenome data sets.

Since the relative abundance of *Methylobacterium* in soybean microbiomes (33–34%) was higher than that in rice microbiomes (8–14%) (Table S3, Fig. S1), the relative abundance of each gene in metagenomes was normalized to the abundance of *Methylobacterium* (Fig. 5B). However, the results obtained were similar to those shown in Fig. 5A, in that the abundance of NMG pathway genes in both sets of soybean microbiomes was higher than that in both sets of rice microbiomes (Fig. 5B).

### Growth using methylamine as a sole carbon source

We determined methylobacterial CFUs (pink colonies) on AMS agar supplemented with methylamine or methanol. The CFU of soybean stems was 8.5×10^5^ g^−1^ tissue on methylamine medium and 8.4×10^5^ g^−1^ tissue on methanol medium (Table S8). The CFU of soybean leaves was 2.0×10^7^ g^−1^ tissue on methylamine medium and 1.9×10^7^ g^−1^ tissue on methanol medium (Table S8). Thus, CFU values were similar between methylamine and methanol media for the stems and leaves of field-grown soybean plants, suggesting that soybean shoots harbored similar numbers of pink-pigmented bacteria (possibly methylobacteria) that were able to grow using methylamine and methanol as the sole carbon source.

We subsequently examined the cultures of these soybean isolates (Fig. S4). Twenty-four isolates from soybean stems and leaves grown on methanol medium grew on methylamine medium (Fig. S4); all these isolates belonged to Group I based on 16S rRNA gene sequences (data not shown). On the other hand, 12 isolates of rice methylobacteria (17) did not grow on methylamine medium (Fig. S5). These results indicate that methylobacteria inhabiting the soybean shoots utilize methylamine as a sole carbon source, thereby supporting the results of functional metagenomics (Fig. 5).

### Metabolism of urea-related compounds

**Fig. 6:**
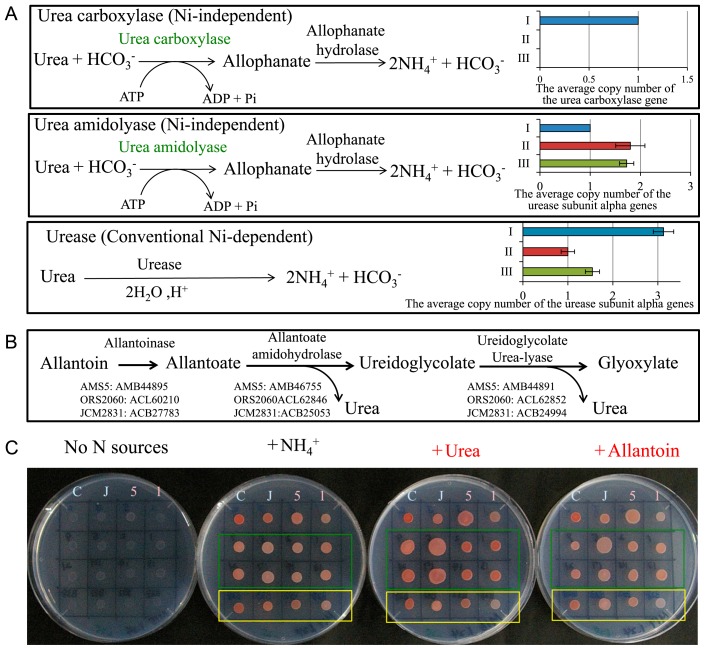
Urea utilization by *Methylobacterium* species. (A) Urea degradation pathways predicted from genome sequences and the average copy number of the relevant genes in each group. (B) Predicted allantoin degradation pathway (64). Accession numbers for the relevant genes are indicated. (C) Growth test without a nitrogen source or with ammonium, urea, or allantoin as the sole nitrogen source for 5 d. 5, *Methylobacterium* sp. AMS5; 1, *M. extorquens* AM1; C, *M. oryzae* CBMB20; and J, *M. radiotolerans* JCM2831. Strains isolated from soybean plants were marked by a green rectangle. Strains S9, S5, S2, and S1 were isolated from the stems of soybean plants and spotted at the upper section from left to right. Strains L21, L17, L14, and L13 were isolated from the leaves of soybean plants and spotted at the lower section from left to right. Strains B38, B33, B32, and B28 were isolated from rice plants and marked by a yellow rectangle and spotted from left to right.

The urea carboxylase gene (AMB46932) was specific to Group I (Table 1); its specificity was supported by strong synteny around the urea carboxylase gene cluster in Group I strains (Fig. S6). Urea carboxylase catalyzes the ATP-dependent and Ni-independent carboxylation of urea, and forms allophanate (34, 56). Allophanate hydrolase uses allophanate to produce two NH_4_^+^ molecules and two HCO_3_^−^ molecules (Fig. 6A). When we searched for genes encoding enzymes associated with urea-related metabolism, including conventional urease, we found genes for two additional enzymes that produce ammonium from urea: Ni-independent urea amidolyase (which generates allophanate) and Ni-dependent urease; these genes were found in all three groups (Fig. 6A, Table S7). Thus, most *Methylobacterium* strains possessed two or three different systems for urea degradation. However, Group I strains appeared to possess a higher number of genes for urea degradation (one copy each of urea carboxylase and urea amidolyase genes, and on average three copies of urease genes) than Group II and III strains (Fig. 6A, Table S7).

In soybean roots, symbiotic rhizobia fix N_2_ into ammonia in the nodules, in which it is assimilated into ureide compounds (allantoin and allantoate), which are then transported to the shoot via xylem (12, 62). Ureide compounds are abundant in soybean tissues (55). Ray *et al.* (55) reported that average ureide concentrations in soybean shoots ranged between 12.4 and 33.1 μmol g^−1^. We surveyed ureide degradation genes (65) in 29 *Methylobacterium* genomes and found that 27 carried genes for complete sets (allantoinase, allantoate amidohydrolase, and ureidoglycorate urea-lyase) for ureide degradation (Fig. 6B, Table S8).

We tested bacterial growth on AMS agar containing NH_4_^+^, urea, or allantoin as the sole nitrogen source (Fig. 6C). Standard strains (AM1, AMS5, JCM2831, and CBMB20) and plant-derived isolates grew well on AMS media supplemented with urea or allantoin. In particular, the growth of AMS5 (Group I) and some Group I isolates from soybean plants appeared to be better in the presence of urea or allantoin than ammonia (Fig. 6C). These results indicate that allantoin and urea are utilized by *Methylobacterium* as nitrogen sources and validate the functions of ureide and urea degradation genes (Fig. 6A and B). As for nitrogen fixation, the *nifH* gene was found exclusively on three genomes (Group II): *M. nodulans* ORS2060, *Methylobacterium* sp. 4–46, and *Methylobacterium* sp. WSM2598, which induce leguminous nodules for symbiotic nitrogen fixation (3, 32). Thus, most methylobacteria do not acquire nitrogen by N_2_ fixation in the phyllosphere environment.

### Group II- and III-specific genes

Group II-specific genes *fixNOPQ* encode cbb3-type cytochrome c oxidase (Table 2, Fig. 3). In rhizobia, this enzyme has high affinity for O_2_ and is required for respiration in symbiotic nitrogen fixation under microaerobic conditions (54). The presence of *fixNOPQ* indicates that the strain, such as *M. nodulans*, belongs to Group II (3, 32) (Fig. 1).

Group III-specific genes *fdh5ABCD* encode a novel formate dehydrogenase because *fdh5* was apparently different from the conventional genes *fdh1–fdh4* in *M. extorquens* AM1 (10). This gene may accelerate energy production during dissimilation in C1 compound metabolism in Group III methylobacteria (Fig. S2).

### Plant colonization by Group I methylobacteria

**Fig. 7:**
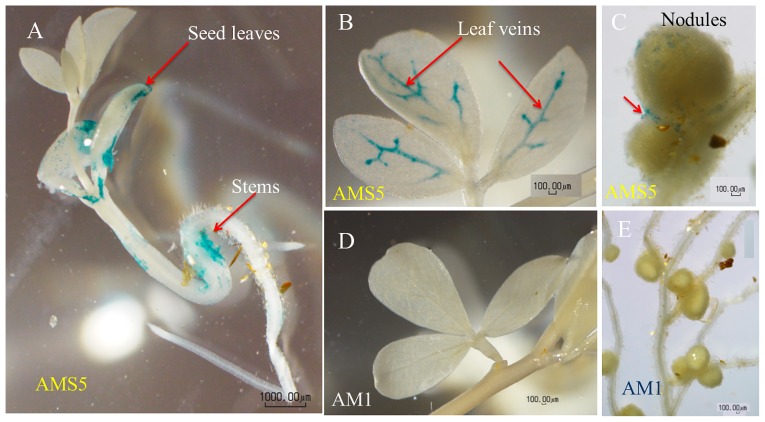
Methylobacterial colonization in *Lotus japonicus* visualized by GUS staining. Plants were inoculated with *GusA*-tagged *Methylobacterium* sp. AMS5 (A–C) or *M. extorquens* AM1 (D, E). (A) Cotyledons and the stem of a 10-d-old seedling. (B) Leaves of a 23-d-old plant. (C, E) Root nodules and (D) leaves of a 40-d-old plant. Scale bars indicate 1,000 μm (A) or 100 μm (B–E).

Group I includes isolates from plants, soil, air, and water (14, 15, 35, 52). We expected plant isolates to carry genes responsible for plant colonization. In order to test this, we inoculated *gusA*-tagged AMS5 (from a soybean plant) and AM1 (from air) into the model legume *L. japonicus*. AMS5 colonized stems, leaves, and root nodules (Fig. 7A, B, and C), and particularly leaf veins (Fig. 7B). The intensity of AMS5 GUS staining was the strongest 23 d after sowing (data not shown). In contrast, the GUS staining of AM1 was hardly observed in leaves or root nodules (Fig. 7D and E). These results suggest that at least AMS5, a soybean-derived *Methylobacterium* strain (2, 44), efficiently colonized *L. japonicus*.

*Methylobacterium* are generally more abundant on aerial than belowground plant parts (16). However, *M. fujisawaense* was isolated from the root nodules of *Lespedeza* (51); therefore, it is not unexpected that AMS5 colonized the root nodules of *L. japonicus* (Fig. 7C).

### Candidate genes for plant colonization

Using CD-HIT, we compared six genomes of Group I strains (AMS5, PA1, AM1, CM4, DM4, and BJ001) and selected genes shared by plant-derived strains (AMS5, PA1, and BJ001). The resultant Venn diagram (Fig. 3B) showed that eight genes were shared among the three strains and many genes were shared between two strains (Table S9). Out of 87 genes shared by the AMS5 and BJ001 genomes, we selected four candidate genes (*csgG*, *tonB*, *pilT*, and the xylose isomerase gene) based on previous findings of plant colonization factors (5, 7, 31, 45) (Table 2). The presence of these four genes in 29 methylobacterial genomes is shown in Table 2 and Table S10.

Although *csgG* was present in all three groups (Table 1), all strains carrying *csgG* were isolated from plants (Table S10). In *E. coli* MC4100, *csgG* is required for the production of curli fimbriae (61), which promote the adhesion of bacteria to plants (31). The synteny of the curli gene cluster was partially conserved between *E. coli* MC4100, *Methylobacterium* sp. AMS5, and *M. populi* BJ001. Thus, the curli protein may play an important role in plant colonization by methylobacteria.

TonB-dependent receptor genes are required for the uptake of iron and carbohydrates, and are also needed for plant colonization by rhizobacteria and for the pathogenicity of plant pathogens (6, 45). High levels of *Sphingomonas* TonB-dependent receptors were detected in the phyllosphere of *A. thaliana* (13). The *tonB* gene (AMB44196) found in CD-HIT (Table 2) was unique to plant-derived methylobacteria, which was different from another major *tonB* gene (AMB44041) that is widely distributed among methylobacterial strains. A quarter of Group I strains and 10% of Group II strains possessed the *tonB* genes (AMB44196) (Table 2).

The *pilT* gene was widely distributed among all three groups (Table 2); the twitching motility protein encoded by this gene is required for the intercellular colonization of plants by the bacterial endophyte, *Azoarcus* sp. BH72 (7). Xylose isomerase is needed to decompose the plant cell wall (5); its genes were only detected in three strains: *Methylobacterium* sp. AMS5 and *M. populi* BJ001 (Group I), and *M. nodulans* ORS 2060 (Group II).

### Abundance of other genes in metagenomes

We evaluated the relative abundance of genes for metabolism and respiration in Group II and III methylobacteria in metagenomic reads of the soybean and rice microbiomes. The relative abundance of *fixNOPQ* and *fdh5ABCD* in rice microbiomes was higher than that in soybean microbiomes (Table 1), which may be explained by the higher abundance of Groups II and III in rice shoots than in soybean plants (Fig. 2A).

The metagenomic abundance of *csgG*, *tonB*, *pilT*, and xylose isomerase genes was higher in soybean microbiomes than in rice microbiomes (Table 2). Since the *tonB* (ABM44196) and xylose isomerase genes were mainly found in Group I strains, their higher abundance in soybean microbiomes may be explained by the dominance of Group I members (Fig. 2A). Although *csgG* and *pilT* were widely distributed in all three groups, their higher abundance in soybean microbiomes than in rice microbiomes indicates their importance for the *Methylobacterium* colonization of soybean plants.

### Ecological and physiological implications of methylamine and ureide metabolism

Although methylamine is produced by the decarboxylation of amino acids and transamination of aldehydes, plant methylamine biosynthesis and methylamine functions in plants are largely unknown (4). The phyllosphere of *A. thaliana* contains 4.78×10^−3^ mM of methylamine (59), whereas the methylamine contents of soybean and rice plants currently remain unknown. Soybean plants may produce more methylamine than rice plants because of their higher nitrogen content (4% N in soybean leaves [28] vs. 1% N in rice shoots [53]). On the other hand, methane emitted from rice paddy fields (11) may be oxidized to methanol by methanotrophic bacteria (50). Thus, rice methylotrophic bacteria may use methanol.

This study also revealed that soybean-associated methylobacteria utilize urea and ureide as a nitrogen source. The exchange of these substrates between soybean plants and methylobacteria may occur in the field. The utilization of ureide compounds (allantoin and allantoinate) by methylobacteria may be parasitic rather than beneficial in that ureide compounds derived from fixed N are competitive between methylobacteria and soybean plants as N sources. In this regard, we found the lack of genes for ureidoglycorate urealyase, the last step of ureide degradation, in *Methylobacterium* sp. strains 4–46 and WSM2598 (Table S8) that nodulate *Lotononis bainesii* (3), suggesting the possible avoidance of metabolic competition for ureide in nodule tissues.

## Supplementary Information



## Figures and Tables

**Table 1:** Group-specific genes involved in metabolism and respiration: distribution among groups and relative abundance in metagenomes.

Group	Accession number^a^	Gene	Function	Distribution within groups (%)^b^			Frequency among 10^6^ metagenome reads			
	
				I	II	III	SoyJp1	SoyJp2	RiceJp1	RiceJp2
I	AMB44789	*mgdD*	*N*-methylglutamate dehydrogenase	100	20	0	83.5	93.7	0	0
	AMB46932		urea carboxylase	100	0	0	96.1	84.1	121.0	2.2

II	ACL57096	*fixN*	cbb3-type cytochrome c oxidase	0	80	0	12.7	10.3	36.2	18.6
	ACL57097	*fixO*	cbb3-type cytochrome c oxidase	0	80	0	0	2.9	45.2	26.8
	ACL57098	*fixP*	cbb3-type cytochrome c oxidase	0	80	0	0	0	58.1	36.2
	ACL57099	*fixQ*	cbb3-type cytochrome c oxidase	0	80	0	8.6	9.7	24.0	6.2

III	ACB23101	*fdh5A*	formate dehydrogenase	63	20	100	4.7	7.0	33.7	28.3
	ACB23100	*fdh5B*	formate dehydrogenase	0	10	100	4.1	4.6	26.6	56.1
	ACB23099	*fdh5C*	formate dehydrogenase	100	80	100	5.3	3.0	36.2	12.5
	ACB23098	*fdh5D*	formate dehydrogenase	100	0	91	5.7	4.3	31.3	12.7

a GenBank accession numbers.

b The percentage of strains of each group carrying each gene.

**Table 2:** Candidate plant association genes in the AMS5 genome and their distribution among groups and abundance in the metagenome.

Accession number^a^	Gene	Function	Distribution within groups (%)^b^			Frequency among 10^6^ metagenome reads			
	
			I	II	III	SoyJp1	SoyJp2	RiceJp1	RiceJp2
AMB43278	*csgG*	curli production assembly protein	25	10	55	103.1	83.3	12.1	3.9
AMB44196	*tonB*	energy transducer periplasmic protein	25	10	0	67.4	61.0	0.0	2.1
AMB44541	*pilT*	twitching motility protein	88	80	91	157.5	209.0	84.3	95.3
AMB44832		xylose isomerase	25	10	0	69.3	69.4	1.2	0.0

a GenBank accession numbers.

b The percentage of strains in each group carrying each gene. Details are shown in Table S6.
