# Supplementary material for: Metagenomic Analysis Revealed Methylamine and Ureide Utilization of Soybean-Associated Methylobacterium
Source: Microbes Environ. 2016 Jul 15;31(3):268–78. doi: 10.1264/jsme2.ME16035 (PMC5017803; doi:10.1264/jsme2.ME16035)
Supplement: Supplementary file 1 [file 31_268_s1.pdf]

Table S1. Bacterial strains and plasmid used in this study

Strain or plasmid	Description[a]	Reference
Strains		
Methylobacterium sp.		
AMS5	Field isolate from soybean stem	3
AMS5g	AMS5 tagged with *gusA* gene	This study
M. extorquens		
AM1	Isolate from air dust	19
AM1g	AM1 tagged with *gusA* gene	This study
Escherichia coli		
DH5	Donor strain for conjugation	
MT616	Helper strain for conjugation	7
Mesorhizobium loti		
MAFF303099	Rhizobia for *Lotus japonicus*	10
Plasmid		
mTn5SS*gusA*20	Plasmid used for transposon insertion, *gusA*, Ap^r, Sm^r, Sp^r	21

[a] Ap^r, ampicillin resistant; Sm^r, streptomycin resistant; Sp^r, spectinomycin resistant.

Table S2. Statistical summary of metagenome data of bacteria associated with soybean and rice.

Sample name	SoyJp1	SoyJp2	RiceJp1	RiceJp2
ID[a]	4558115.3	4558119.3	4450454.3	4450453.3
Total number of sequence reads	524,958	467,744	803,190	551,996
Total sequence length (bp)	274,622,507	248,105,638	327,547,777	240,215,335
Average sequence length (bp)	523	530	407	435
Average GC content	66%	66%	64%	62%
α-Diversity	97	99	375	379
Plant[b]	Soybean	Soybean	Rice	Rice
Cultivar	Enrei	Enrei	Nipponbare (Japonica type)	Kasalath (Indica type)
Parts of plant	Stems	Stems	Shoots	Shoots
Country	Japan	Japan	Japan	Japan
Reference	This study	This study	18	18

[a] ID was assigned by the metagenomic data MG-RAST server (15).

[b] Bacterial communities of SoyJp1/SoyJp2 and RiceJp1/RiceJp2 were extracted from soybean stems and rice shoots, respectively. Soybean and rice plants were cultivated at the same field (Kashimadai) in Japan (see text).

Table S3. Phylogenetic compositions of bacterial communities associated with soybean and rice

	Relative abundance (%)[a]			
Taxonomic group	SoyJp1	SoyJp2	RiceJp1	RiceJp2
Bacteria	92.67	92.68	89.57	88.88
Actinobacteria	21.06	21.05	16.93	12.92
Alphaproteobacteria	61.44	61.56	52.08	49.91
Rhizobiales	55.85	56.06	37.53	31.27
Methylobacterium	33.33	33.62	13.52	7.91
Agrobacterium	5.25	5.27	2.27	3.26
Rhizobium	2.83	2.81	4.14	4.89
Bradyrhizobium	2.09	2.07	2.65	1.92
Gammaproteobacteria	6.4	6.34	10.27	11.4
Betaproteobacteria	2.37	2.34	4.99	10.15

[a] Relative abundance of each phylogenetic groups in metagenomes.

Table S4. The list of genomes used for identifying gene distribution

Group	Strain	Isolation source	Assembly status	Accession number[a]	Reference
I	*Methylobacterium* sp. AMS5	Soybean	Complete	CP006992-CP006995	16
I	*M. extorquens* AM1	Air	Complete	CP001510-CP001514	20
I	*M. extorquens* PA1	*A. thaliana*	Complete	CP000908	14
I	*M. extorquens* CM4	Soil	Complete	CP001298-CP001300	14
I	*M. extorquens* DM4	Soil	Complete	FP103042-FP103044	20
I	*M. extorquens* DSM13060	Pine tree	Draft	AGJK00000000	11
I	*M. populi* BJ001	Popular	Complete	CP001029-CP001031	14
I	*Methylobacterium* sp. MB200	Biogas reacter	Draft	KB316282-KB316287	
II	*M. nodulans* ORS 2060	*C. podocarpa*	Complete	CP001349-CP001356	14
II	*Methylobacterium* sp. 4-46	*L. bainesii*	Complete	CP000943-CP000945	14
II	*Methylobacterium* sp. WSM2598	*L. bainesii*	Draft	KB900609-KB900613	4
II	*M. aquaticum* 22A	Moss	Complete	AP014704-AP014709	1
II	*M. aquaticum* DSM16371	Water	Draft	LABX01000001-LABX01000512	
II	*M. tarhaniae* DSM 25844	Soil	Draft	NZ_LABZ01000001-NZ_LABZ01000483	
II	*M. variabile* DSM16961	Water	Draft	LABY01000001-LABY01000562	
II	*M. platani* JCM14648	Platanus	Draft	BBDG01000001-BBDG01002268	
II	M. platani SE3.6	Rice	Draft	JTHG01000001-JTHG01000560	
II	M. platani SE2.11	Rice	Draft	JTHF01000001-JTHF01000506	
III	*M. radiotolerans* JCM2831	Rice	Complete	CP00100-CP001009	14
III	*M, radiotolerans* 78c	Pumpkin	Draft	NZ_JXTO01000001-NZ_JXTO01000271	5
III	*M. oryzae* CBMB20[n]	Rice	Complete	CP003811	12
III	*Methylobacterium* sp. EUR3	Unknown	Draft	NZ_JAGD01000001-NZ_JAGD01000007	
III	*M. mesophilicum* SR1.6/6	Orange	Draft	ANPA01000001-ANPA01000029	2
III	*Methylobacterium* sp. L2-4	*Jatropha*	Draft	AVNX01000001-AVNX01000382	13
III	*Methylobacterium* sp.	Unknown	Draft	KB911270-KB911364	
III	*Methylobacterium* sp. B34	Unknown	Draft	BADE01000001-BADE01001622	
III	*Methylobacterium* sp. B1	Unknown	Draft	BACT01000001-BACT01001090	
III	*Methylobacterium* sp. ME121	Soil	Draft	BBUX01000001-BBUX01000197	8
III	*Methylobacterium* sp. UNCCL110	Unknown	Draft	JQJG01000001-JQJG01000142	
Others	*Methylobacterium* sp. ARG-1	Mashroom	Draft	LHCD01000001-LHCD01000092	
Others	*Methylobacterium* sp. 10	Unknown	Draft	JAEV01000001-JAEV01000010	
Others	*Methylobacterium* sp. 88A	water	Draft	AQVT01000001-AQVT01000004	
Others	*Methylobacterium* sp. 77	Unknown	Draft	ARCS01000001-ARCS01000002	
Others	*Methylobacterium* sp. GXF4	Grape	Draft	AKFK01000001-AKFK01000123	9

[a] The accession number is GenBank accession number.

Table S5. Replicons and general features of complete genomes of *Methylobacterium*

Group	Strain	Replicon	Size(bp)	GC content	CDS[a]	rRNA operons	tRNA[b]
I	*Methylobacterium* sp.	Chromosome	5,435,450	68.4%	4963	5	58
	Methylobacterium sp.	pAMS5a	117,697	65.3%	160	0	2
	Methylobacterium sp.	pAMS5b	25,608	65.3%	28	0	0
	Methylobacterium sp.	pAMS5c	20,451	66.3%	27	0	0
		Total	5,599,206	68.4%	5178	5	60
I	*M. extorquens* AM1	Chromosome	5,511,322	68.7%	4947	5	57
	M. extorquens AM1	Megaplasmid	1,261,460	67.6%	1161	0	6
	M. extorquens AM1	p1META1	44,195	67.9%	33	0	0
	M. extorquens AM1	p2META1	37,858	65.2%	35	0	0
	M. extorquens AM1	p3META1	24,943	66.9%	30	0	0
		Total	6,879,778	68.5%	6206	5	63
I	*M. extorquens* PA1	Chromosome	5,471,154	68.2%	4829	5	58
I	*M. extorquens* CM4	Chromosome	5,777,908	68.2%	5173	5	62
	M. extorquens CM4	pMCHL01	380,207	66.3%	306	0	0
	M. extorquens CM4	pMCHL02	22,617	63.9%	37	0	0
		Total	6,180,732	68.1%	5516	5	62
I	*M. extorquens* DM4	Chromosome	5,943,768	68.1%	5593	5	58
	M. extorquens DM4	p1METDI	141,504	65.3%	105	0	0
	M. extorquens DM4	p2METDI	38,579	63.7%	37	0	0
		Total	6,123,851	68.0%	5735	5	58
I	*M. populi* BJ001	Chromosome	5,800,441	69.4%	5314	5	56
	M. populi BJ001	pMPOP01	25,164	64.9%	25	0	0
	M. populi BJ001	pMPOP02	23,392	66.8%	26	0	0
		Total	5,848,997	69.4%	5365	5	56
II	*M. nodulans* ORS2060	Chromosome	7,772,460	68.9%	7355	7	70
	M. nodulans ORS2060	pMNOD01	487,734	65.9%	402	0	2
	M. nodulans ORS2060	pMNOD02	458,070	65.7%	409	0	0
	M. nodulans ORS2060	pMNOD03	40,463	64.2%	53	0	0
	M. nodulans ORS2060	pMNOD04	37,542	61.6%	48	0	0
	M. nodulans ORS2060	pMNOD05	20,286	61.4%	21	0	0
	M. nodulans ORS2060	pMNOD06	12,638	60.5%	13	0	0
	M. nodulans ORS2060	pMNOD07	9,829	67.2%	7	0	0
		Total	8,839,022	68.4%	8308	7	72
II	*Methylobacterium* sp. 4-46	Chromosome	7,659,055	71.6%	6609	6	65
	Methylobacterium sp. 4-46	pM44601	57,951	65.1%	62	0	0
	Methylobacterium sp. 4-46	pM44602	20,019	59.2%	13	0	0

		Total	7,737,025	71.5%	6684	6	65
II	*M. aquaticum* 22A	Chromosome	5,348,274	71.1%	5074	10	84
	M. aquaticum 22A	pMaq22A-1	1,571,989	70.9%	1514	1	13
	M. aquaticum 22A	pMaq22A-2	462,889	67.5%	480	0	0
	M. aquaticum 22A	pMaq22A-3	85,702	67.8%	108	0	0
	M. aquaticum 22A	pMaq22A-4	50,170	70.3%	68	0	0
	M. aquaticum 22A	pMaq22A-5	38,936	66.7%	44	0	0
		Total	7,557,960		7288	11	97
III	*M. radiotolerans* JCM2831	Chromosome	6,077,833	71.5%	5686	4	55
	M. radiotolerans JCM2831	pMRAD01	586,164	69.6%	507	0	1
	M. radiotolerans JCM2831	pMRAD02	47,003	62.5%	52	0	0
	M. radiotolerans JCM2831	pMRAD03	42,985	63.2%	43	0	1
	M. radiotolerans JCM2831	pMRAD04	37,743	63.7%	41	0	1
	M. radiotolerans JCM2831	pMRAD05	36,410	62.0%	31	0	0
	M. radiotolerans JCM2831	pMRAD06	27,836	61.0%	23	0	1
	M. radiotolerans JCM2831	pMRAD07	22,114	61.1%	21	0	0
	M. radiotolerans JCM2831	pMRAD08	21,022	65.1%	27	0	0
		Total	6,899,110	71.0%	6431	4	59
III	*M. oryzae* CBMB20	Chromosome	6,286,629	69.8%	6274	4	57

[a] The number of CDSs was counted based on CDSs extracted from GenBank files by GenomeMatcher software (17).

[b] The number of tRNA was based on the NCBI database.

Table. S6. Colony forming unit (CFU) of stems and leaves of field-grown soybean plants

Sample	CFU (10^5 /g tissue)		Ratio[a] (%)
	Methyamine (MA)	Methanol (MeOH)	
Stem	8.5 ± 4.4	8.4 ± 2.9	107 ± 16
Leaf	199 ± 51	189 ± 46	105 ± 4

Macerated soybean tissues were serially diluted with sterilized water, and plated on AMS agar media supplemented with 20 mM of methylamine or methanol. The plates were incubated at 28 °C. CFU was obtained 6 days after inoculation.

[a] The ratio of CFU from the methylamine medium to that from methanol medium.

Table S7. Copy number of genes for degradation of urea

Group	Taxon	Strain	Copy number of genes	
			Urease subunit alpha[a]	Urea amidolyase[b]
I	*Methylobacterium* sp.	AMS5	3	1
	M. extorquens	AM1	3	1
	M. extorquens	PA1	3	1
	M. extorquens	CM4	3	1
	M. extorquens	DM4	4	1
	M. extorquens	DSM13060	2	1
	M. populi	BJ001	3	1
	Methylobacterium sp.	MB200	4	1
II	*M. nodulans*	ORS2060	1	2
	Methylobacterium sp.	4-46	1	1
	Methylobacterium sp.	WSM2598	0	0
	M. aquaticum	22A	1	2
	M. aquaticum	DSM16371	1	2
	M. tarhaniae	DSM25844	1	1
	M. variabile	DSM16961	1	3
	M. platani	JCM14648	2	2
	M. platani	SE3.6	1	3
	M. platani	SE2.11	1	2
III	*M. radiotolerans*	JCM2831	2	2
	M. radiotolerans	78c	2	2
	M. oryzae	CBMB20	1	2
	Methylobacterium sp.	EUR 3 AL-11	2	2
	M. mesophilicum	SR1.6/6	1	1
	Methylobacterium sp.	L2-4	2	2
	Methylobacterium sp.	285MFTsu5.1	2	2
	Methylobacterium sp.	B34	1	2
	Methylobacterium sp.	B1	1	1
	Methylobacterium sp.	ME121	2	2
	Methylobacterium sp.	UNCCL110	1	1

[ab] Accession numbers for query sequences were AMB46158 (Alpha subunit of urease) and AMB44668 (Urea amidolyase) in *Methylobacterium* sp. AMS5 (16).

Table S8. Copy number of genes for degradation of allantoin

Group	Taxon	Strain[a]	Copy number of genes<br>Allantoinase[a]	Allantoate amidohydrolase[b]	Ureidoglycolate Urea-lyase[c]
I	*Methylobacterium* sp.	AMS5	1	2	1
	M. extorquens	AM1	1	2	1
	M. extorquens	PA1	1	4	1
	M. extorquens	CM4	1	3	1
	M. extorquens	DM4	1	2	1
	M. extorquens	DSM13060	1	2	1
	M. populi	BJ001	1	1	1
	Methylobacterium sp.	MB200	1	1	1
II	*M. nodulans*	ORS2060	1	3	1
	Methylobacterium sp.	4-46	3	5	0
	Methylobacterium sp.	WSM2598	3	4	0
	M. aquaticum	22A	2	6	1
	M. aquaticum	DSM16371	2	6	1
	M. tarhaniae	DSM25844	1	4	1
	M. variabile	DSM16961	2	6	1
	M. platani	JCM14648	1	7	1
	M. platani	SE3.6	4	4	1
	M. platani	SE2.11	3	4	2
III	*M. radiotolerans*	JCM2831	2	6	1
	M. radiotolerans	78c	1	3	1
	M. oryzae	CBMB20	1	7	1
	Methylobacterium sp.	EUR3AL-11	2	5	1
	M. mesophilicum	SR1.6/6	1	5	1
	Methylobacterium sp.	L2-4	2	5	1
	Methylobacterium sp.	285MFTsu5.1	2	6	1
	Methylobacterium sp.	B34	1	3	1
	Methylobacterium sp.	B1	1	3	1
	Methylobacterium sp.	ME121	2	5	1
	Methylobacterium sp.	EUR3AL-11	2	5	1

[abc] The accession numbers for query gene were KIU28859.1 (Allantoinase), AMB46755 (Allantoate amidohydrolase) and AMB44891 (Ureidoglycolate Urea-lyase).

Table S9. The list of genes specific to Group I strains isolated from plants

Strains	Locus tag[a]	Function
AMS5, PA1, BJ001	Y590_03720	urease accessory protein UreE
AMS5, PA1, BJ001	Y590_03890	XRE family transcriptional regulator
AMS5, PA1, BJ001	Y590_11095	regulatory protein RecX
AMS5, PA1, BJ001	Y590_12375	hypothetical protein
AMS5, PA1, BJ001	Y590_12690	hypothetical protein
AMS5, PA1, BJ001	Y590_13270	hypothetical protein
AMS5, PA1, BJ001	Y590_17145	ABC transporter substrate-binding protein
AMS5, PA1, BJ001	Y590_23380	hypothetical protein
AMS5, PA1	Y590_00635	transposase IS4
AMS5, PA1	Y590_01480	ABC transporter permease
AMS5, PA1	Y590_02440	flagellin
AMS5, PA1	Y590_02760	transposase
AMS5, PA1	Y590_04685	hypothetical protein
AMS5, PA1	Y590_04750	hypothetical protein
AMS5, PA1	Y590_04900	integrase
AMS5, PA1	Y590_09685	hypothetical protein
AMS5, PA1	Y590_10605	autolysin
AMS5, PA1	Y590_14590	cold-shock protein
AMS5, PA1	Y590_16310	transposase
AMS5, PA1	Y590_18710	hypothetical protein
AMS5, PA1	Y590_19275	glutathione S-transferase
AMS5, PA1	Y590_19280	dTDP-4-dehydrorhamnose reductase
AMS5, PA1	Y590_19285	UDP-glucose 4-epimerase
AMS5, PA1	Y590_19290	UDP-N-acetylglucosamine 2-epimerase
AMS5, PA1	Y590_19875	ABC transporter ATP-binding protein
AMS5, PA1	Y590_23870	hypothetical protein
AMS5, BJ001	Y590_00075	hypothetical protein
AMS5, BJ001	Y590_00080	lytic transglycosylase
AMS5, BJ001	Y590_00085	curli production assembly protein CsgG
AMS5, BJ001	Y590_00115	hypothetical protein
AMS5, BJ001	Y590_00150	malonate transporter
AMS5, BJ001	Y590_00155	malonate carrier protein
AMS5, BJ001	Y590_00250	hypothetical protein
AMS5, BJ001	Y590_01365	FkbM family methyltransferase
AMS5, BJ001	Y590_01545	hypothetical protein
AMS5, BJ001	Y590_02895	hypothetical protein

AMS5, BJ001	Y590_02900	peptidase C14
AMS5, BJ001	Y590_02905	hypothetical protein
AMS5, BJ001	Y590_02915	hypothetical protein
AMS5, BJ001	Y590_02920	hypothetical protein
AMS5, BJ001	Y590_02925	hypothetical protein
AMS5, BJ001	Y590_02930	hypothetical protein
AMS5, BJ001	Y590_02935	membrane protein
AMS5, BJ001	Y590_02940	hypothetical protein
AMS5, BJ001	Y590_02945	hypothetical protein
AMS5, BJ001	Y590_02965	hypothetical protein
AMS5, BJ001	Y590_03170	NAD-dependent dehydratase
AMS5, BJ001	Y590_03700	MxaD
AMS5, BJ001	Y590_03940	lipolytic protein g-d-s-l family
AMS5, BJ001	Y590_04765	biopolymer transporter
AMS5, BJ001	Y590_04770	cell envelope biogenesis protein TonB
AMS5, BJ001	Y590_06535	transcription factor
AMS5, BJ001	Y590_06540	twitching motility protein PilT
AMS5, BJ001	Y590_07380	hypothetical protein
AMS5, BJ001	Y590_07905	XRE family transcriptional regulator
AMS5, BJ001	Y590_07975	hypothetical protein
AMS5, BJ001	Y590_07980	membrane protein
AMS5, BJ001	Y590_07985	LysR family transcriptional regulator
AMS5, BJ001	Y590_07990	glucose-fructose oxidoreductase
AMS5, BJ001	Y590_08000	xylose isomerase
AMS5, BJ001	Y590_08005	major facilitator transporter
AMS5, BJ001	Y590_08010	3-oxoacyl-ACP reductase
AMS5, BJ001	Y590_08015	dehydratase
AMS5, BJ001	Y590_08020	3D-(3,5/4)-trihydroxycyclohexane-1,2-dionehydrolase
AMS5, BJ001	Y590_08025	hypothetical protein
AMS5, BJ001	Y590_08030	2,4-dihydroxyhept-2-ene-1,7-dioic acid aldolase
AMS5, BJ001	Y590_08035	hypothetical protein
AMS5, BJ001	Y590_08040	hypothetical protein
AMS5, BJ001	Y590_08050	tat pathway signal sequence
AMS5, BJ001	Y590_08055	GMC family oxidoreductase
AMS5, BJ001	Y590_08060	alcohol dehydrogenase
AMS5, BJ001	Y590_08065	hypothetical protein
AMS5, BJ001	Y590_08070	chemotaxis sensory transducer
AMS5, BJ001	Y590_08075	rhizopine-binding protein

AMS5, BJ001	Y590_08320	hypothetical protein
AMS5, BJ001	Y590_08325	hypothetical protein
AMS5, BJ001	Y590_08330	glycosyl transferase
AMS5, BJ001	Y590_08340	adenylate cyclase
AMS5, BJ001	Y590_09110	metallophosphoesterase
AMS5, BJ001	Y590_10110	peptide ABC transporter substrate-bindingprotein
AMS5, BJ001	Y590_10345	MarR family transcriptional regulator
AMS5, BJ001	Y590_10630	MFS transporter
AMS5, BJ001	Y590_10955	transposase
AMS5, BJ001	Y590_11475	histidine kinase
AMS5, BJ001	Y590_12215	hypothetical protein
AMS5, BJ001	Y590_13245	glutathione S-transferase
AMS5, BJ001	Y590_13335	hypothetical protein
AMS5, BJ001	Y590_13882	hypothetical protein
AMS5, BJ001	Y590_14305	hypothetical protein
AMS5, BJ001	Y590_14780	hypothetical protein
AMS5, BJ001	Y590_15543	hypothetical protein
AMS5, BJ001	Y590_15820	LysR family transcriptional regulator
AMS5, BJ001	Y590_16810	hypothetical protein
AMS5, BJ001	Y590_17495	hypothetical protein
AMS5, BJ001	Y590_17500	flagellin hook protein
AMS5, BJ001	Y590_17505	AraC family transcriptional regulator
AMS5, BJ001	Y590_18135	hypothetical protein
AMS5, BJ001	Y590_18175	hypothetical protein
AMS5, BJ001	Y590_18180	hypothetical protein
AMS5, BJ001	Y590_18185	hypothetical protein
AMS5, BJ001	Y590_19330	hypothetical protein
AMS5, BJ001	Y590_19370	response regulator receiver
AMS5, BJ001	Y590_20550	hypothetical protein
AMS5, BJ001	Y590_21405	hypothetical protein
AMS5, BJ001	Y590_22045	hypothetical protein
AMS5, BJ001	Y590_22055	MobB mobilization protein
AMS5, BJ001	Y590_23320	hypothetical protein
AMS5, BJ001	Y590_24790	hypothetical protein
AMS5, BJ001	Y590_25790	plasmid stabilization protein
AMS5, BJ001	Y590_25795	addiction module antitoxin
AMS5, BJ001	Y590_25800	chromosome partitioning protein ParA
AMS5, BJ001	Y590_25805	hypothetical protein

AMS5, BJ001	Y590_25905	metallophosphoesterase
PA1, BJ001	Mext_0357	hypothetical protein
PA1, BJ001	Mext_0502	phosphoglycerate mutase
PA1, BJ001	Mext_0508	group 1 glycosyl transferase
PA1, BJ001	Mext_0634	lytic transglycosylase
PA1, BJ001	Mext_0843	XRE family transcriptional regulator
PA1, BJ001	Mext_1356	cytochrome c class I
PA1, BJ001	Mext_1725	hypothetical protein
PA1, BJ001	Mext_1879	hypothetical protein
PA1, BJ001	Mext_1880	regulatory protein Crp
PA1, BJ001	Mext_1881	PAS sensor protein
PA1, BJ001	Mext_1884	hypothetical protein
PA1, BJ001	Mext_2335	hypothetical protein
PA1, BJ001	Mext_2746	hypothetical protein
PA1, BJ001	Mext_2756	death-on-curing family protein
PA1, BJ001	Mext_2929	prevent-host-death family protein
PA1, BJ001	Mext_2992	hypothetical protein
PA1, BJ001	Mext_3096	glycyl-tRNA synthetase subunit beta
PA1, BJ001	Mext_3520	hypothetical protein
PA1, BJ001	Mext_3909	addiction module antitoxin
PA1, BJ001	Mext_3973	hypothetical protein
PA1, BJ001	Mext_3974	Fis family transcriptional regulator
PA1, BJ001	Mext_4484	hypothetical protein
PA1, BJ001	Mext_4886	TonB-dependent siderophore receptor

[a] Locus tags of AMS5 and PA1 were shown as representatives. Y590 and Mext were locus tags of strains AMS5 and BJ001, respectively.

Table S10. Distributions of candidate genes for plant colonization

Group	Taxon	Strain[a]	Gene distributions			
			csgG	*tonB*	*pilT*	*Xylose isomerase*
I	*Methylobacterium* sp.	AMS5*	+	+	+	+
	M. extorquens	PA1*	-	-	+	-
	M. extorquens	DSM13060	-	-	-	-
	M. populi	BJ001*	+	+	+	+
	M. extorquens	DM4*	-	-	+	-
	M. extorquens	AM1*	-	-	+	-
	M. extorquens	CM4*	-	-	+	-
	Methylobacterium sp.	MB200	-	-	+	-
II	*M. nodulans*	ORS2060*	-	-	+	+
	Methylobacterium sp.	WSM2598	-	+	-	-
	M. platani	SE3.6	-	-	+	-
	M. platani	SE2.11	-	-	+	-
	M. platani	JCM14648	-	-	+	-
	Methylobacterium sp.	4-46*	+	-	-	-
	M. aquaticum	22A*	-	-	+	-
	M. aquaticum	DSM16371	-	-	+	-
	M. tarhaniae	DSM25844*	-	-	+	-
	M. variabile	DSM16961	-	-	+	-
III	*M. radiotolerans*	JCM2831*	-	-	+	-
	M. radiotolerans	78c	-	-	+	-
	M. mesophilicum	SR1.6/6	+	-	+	-
	Methylobacterium sp.	L2-4	+	-	+	-
	Methylobacterium sp.	285MFTsu5.1	+	-	+	-
	Methylobacterium sp.	B34	-	-	+	-
	M. oryzae	CBMB20*	+	-	+	-
	Methylobacterium sp.	UNCCL110	+	-	-	-
	Methylobacterium sp.	B1	+	-	+	-
	Methylobacterium sp.	ME121	-	-	+	-
	Methylobacterium sp.	EURAL-11	-	-	+	-

[a] Strains filled with gray were isolated from plants. Asterisk shows that the complete genome sequences were determined.

[b] "+" and "–" indicate the presence and absence of gene.

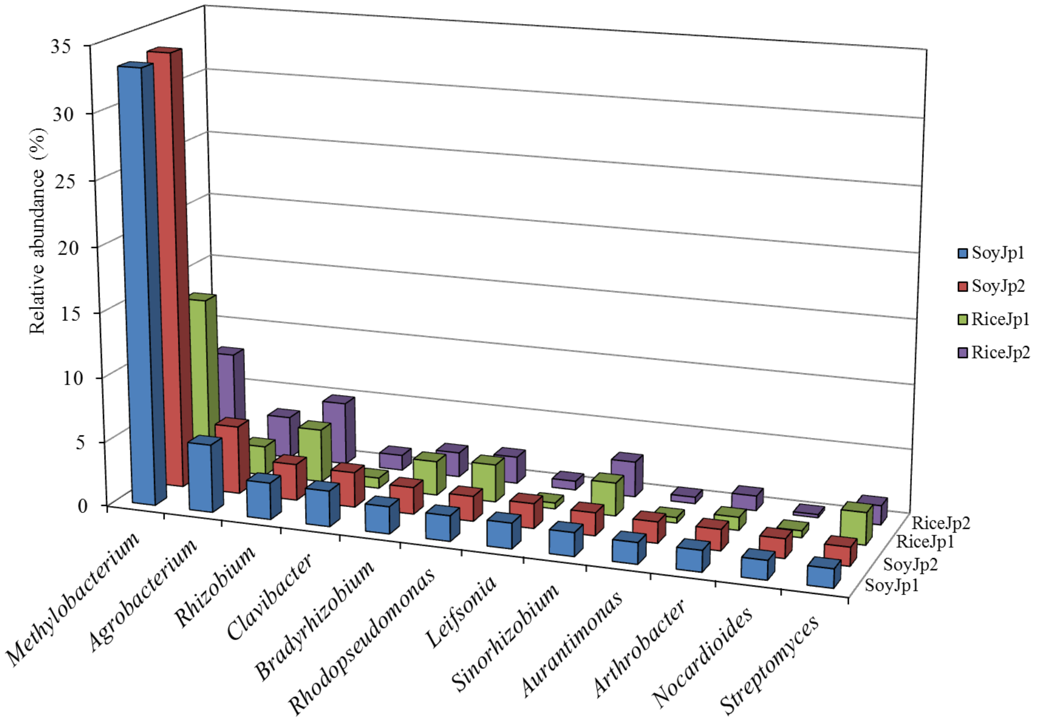

Fig. S1. The genus level phylogenetic composition of bacterial community associated with soybean and rice. Top twelve abundant genus of bacteria associated with soybean and rice plants are shown. More higher level phylogenetic composition is shown at Table S3.

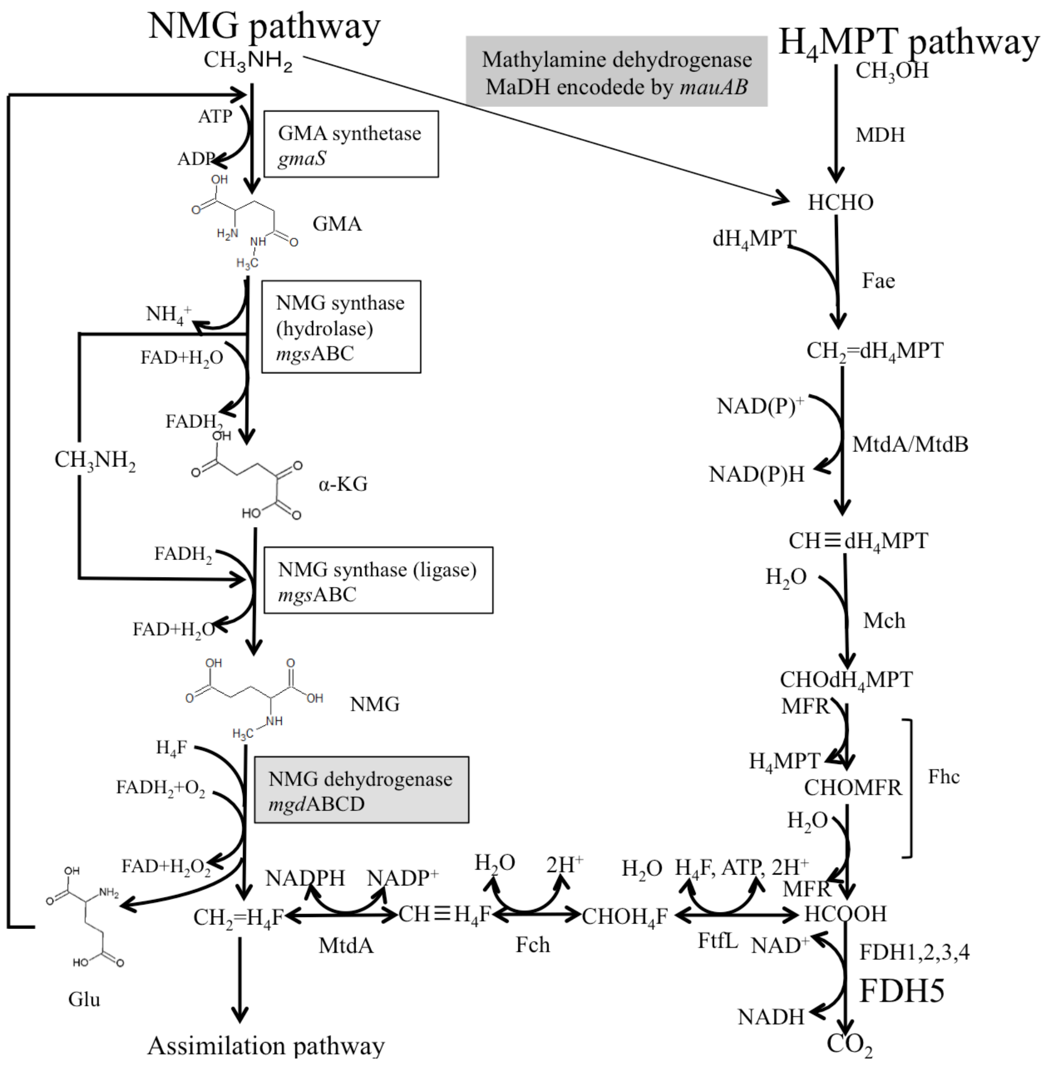

Fig. S2. Metabolic pathways of methylamine and methanol in *Methylobacterium*. GMA, γ-glutamylmethylamide; α-KG, α-ketoglutarate; NMG, *N*-methylglutamate; Glu, Glutamate; dH_4MPT, dephosphotetrahydromethanopterin; CH2=dH_4MPT, methylene- dH_4MPT; CH≡dH_4MPT, methenyl-dephosphoH_4MPT; CHOdH_4MPT, formyl dH_4MPT; MFR, methanofuran; CHOMFR, formyl-MFR; H_4F, tetrahydrofolate; CHOH_4F, formyl-H_4F; CH≡H_4F, methenyl-H_4F; MDH, methanol dehydrogenase; Fae, formaldehyde activating enzyme; MtdA and MtdB, methylene-tetrahydromethanopterin dehydrogenase; Mch, methenyl-dH_4MPT cyclohydrolase; Fhc, formyltransferase/hydrolase complex; fdh, formate dehydrogenase; FtfL, formate-tetrahydrofolae ligase; Fch, methenyl-H_4F cyclohydrolase.

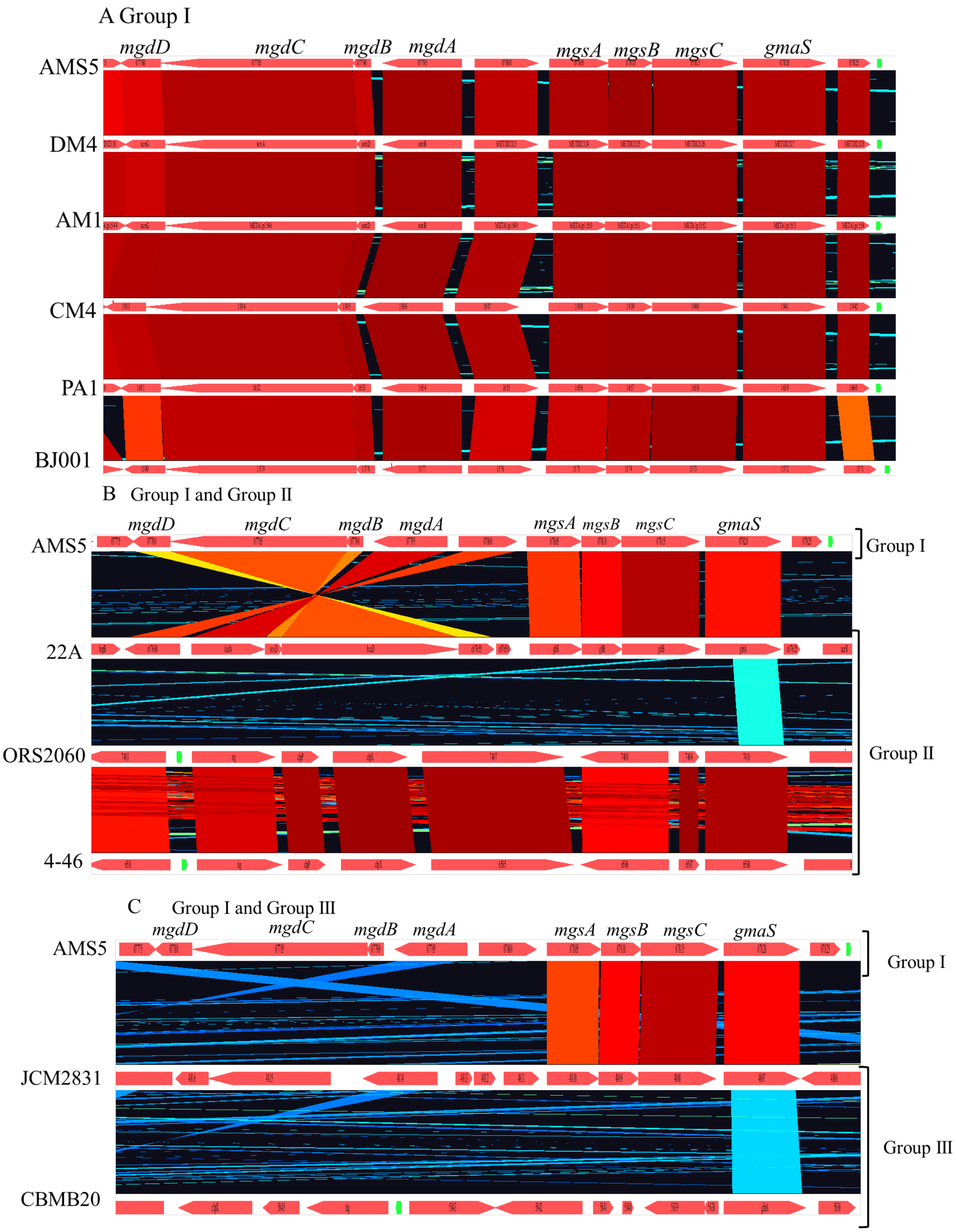

Fig. S3. Chromosomal synteny of the methylamine utilization gene region in *Methylobacterium* species. (A) Comparison among Group I strains. (B) Comparison among strains from Groups I and II. (C) Comparison among strains from Groups I and III. The alignment of sequences and similarity search were conducted by GenomeMatcher (17) with blastp. The genes gmaS, mgsABC, and mgdABCD encode GMA synthetase, NMG synthase, and NMG dehydrogenase, respectively.

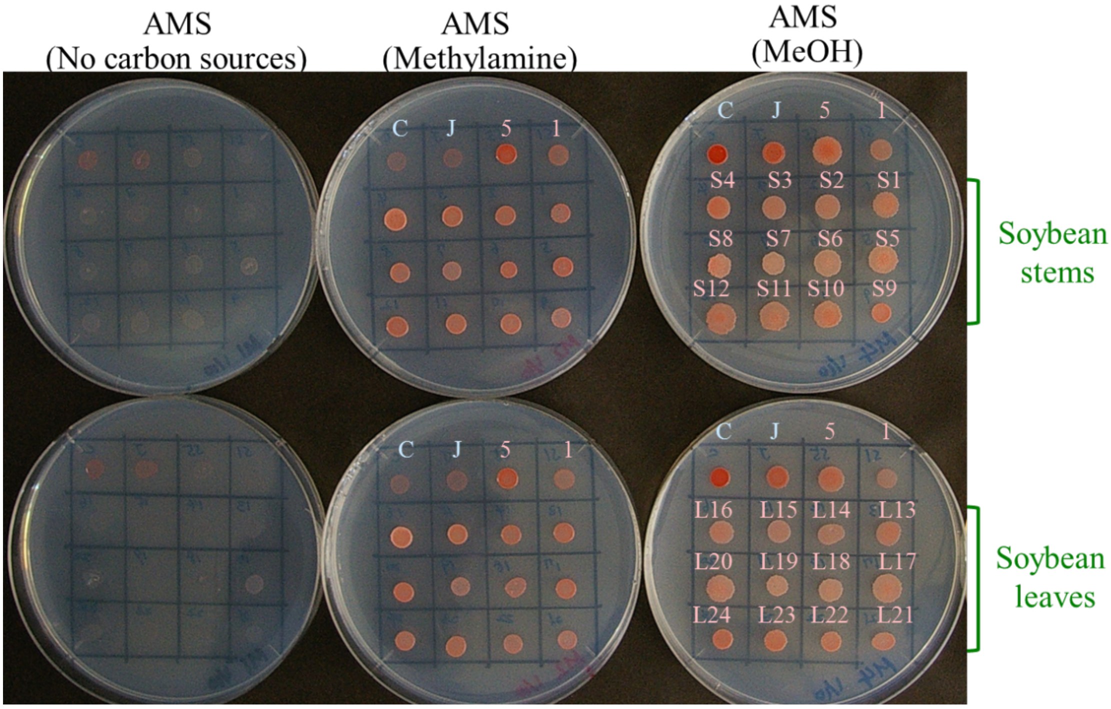

Fig. S4. Growth test of methylobacterial strains isolated from soybean for 12 days. 1, *M. extorquens* AM1; 5, *Methylobacterium* sp. AMS5; J, *M. radiotolerans* JCM2831; C, *M. oryzae* CBMB20. S1 to S12 were isolates from soybean leaves and L13 to L24 were isolates from soybean stems using methanol as a sole carbon source.

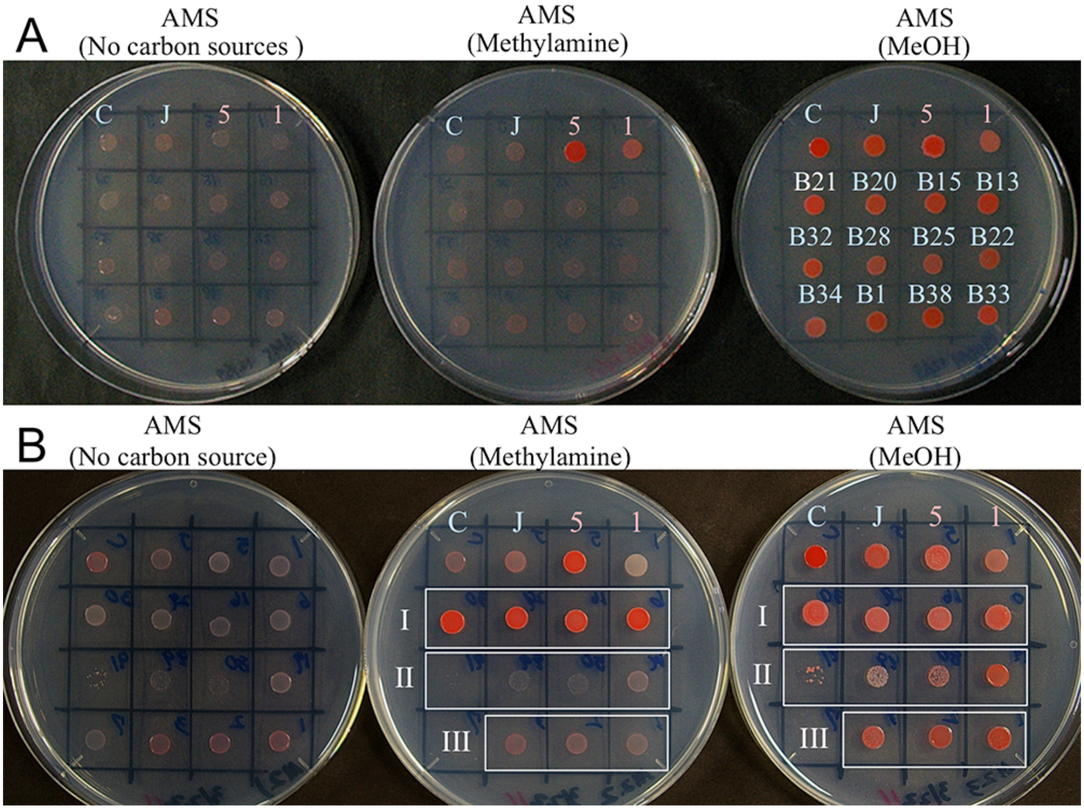

Fig. S5. Growth test of methylobacterial strains isolated from rice (6) (Panel A) and soybean plants (3) (Panel B) for 7 days. 1, *M. extorquens* AM1; 5, *Methylobacterium* sp. AMS5; J, *M. radiotolerans* JCM2831; C, *M. oryzae* CBMB20. (A) B13 to 34 were isolates from shoots of rice plants (6). B13 to 34 were group III strains except B21 written in white on the basis of 16S rRNA gene sequences. (B) Strains isolated from soybean stems (3) were marked by Groups I, II and III based on phylogenetic positions (3) (Fig. 1). Strains AMS30, AMS29, AMS16 and AMS6 (M4 in Group I), strains MM91, AMS99, AMS80 and AMS19 (M5 in Group II), strains AMS3, AMS2 and AMS1 (M2 in Group III) were spotted from left to right. Note that Group I strains exclusively grew on AMS medium with methylamine, while Group II and III strains did not (Panel B).

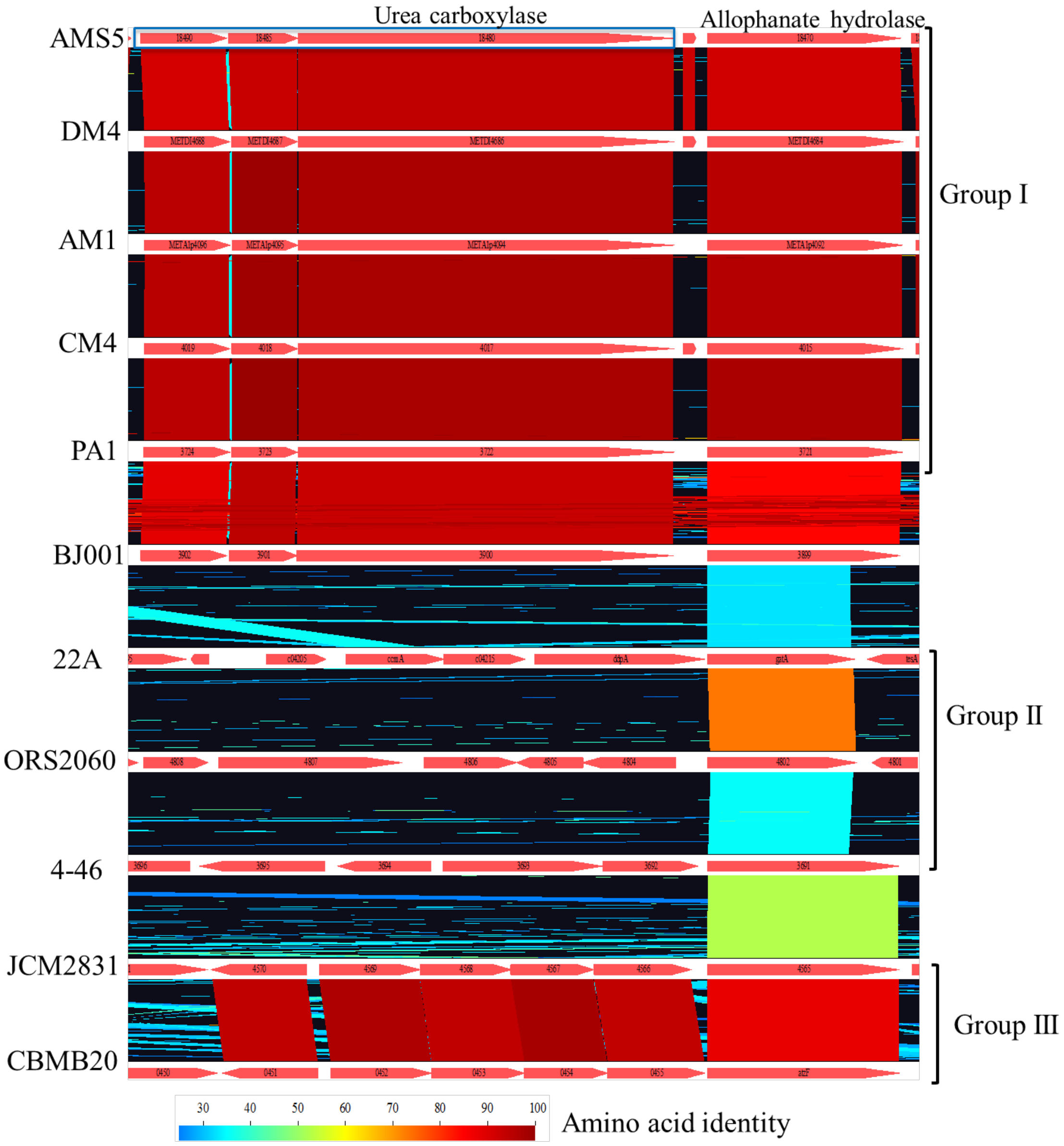

Fig. S6. Chromosomal synteny of the urea carboxylase and allophanate hydrolase genes region in *Methylobacterium* species. The alignment of sequences and similarity search were conducted by GenomeMatcher (17).

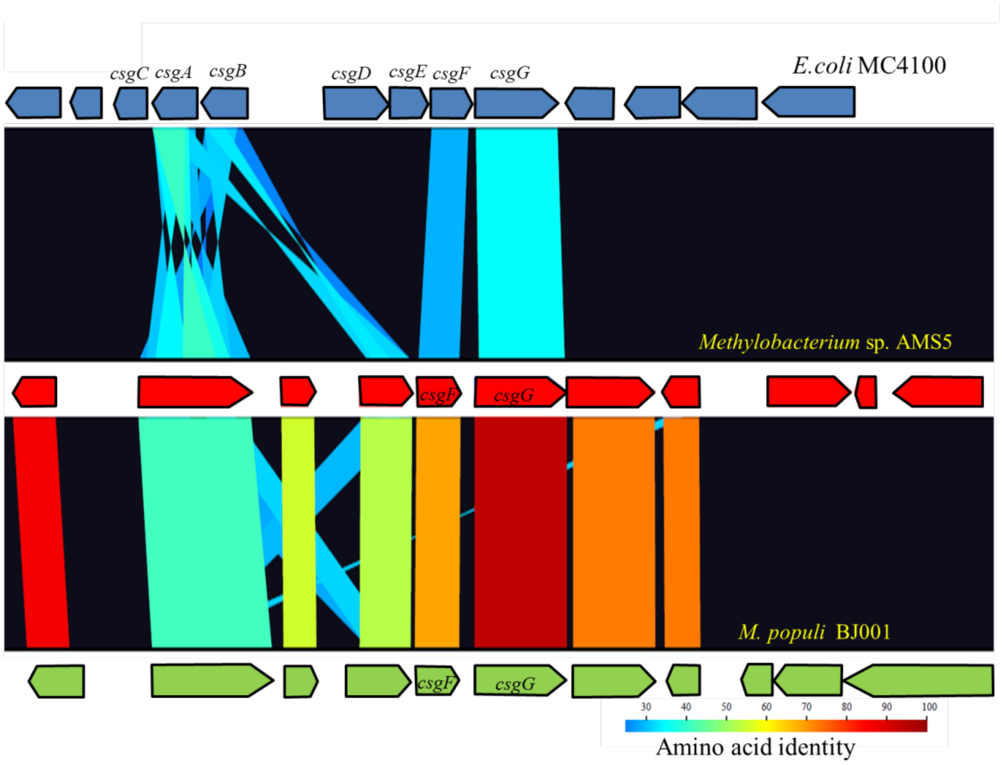

Fig. S7. Comparison of curli fimbriae gene regions among *Escherichia coli* MC4100 and *Methylobacterium* species. The alignment of sequences and similarity search were conducted by GenomeMatcher (17).

References

1. Akio, T., Y. Ogura, T. Hayashi, and K. Kimbara. 2015. Complete genome sequence of *Methylobacterium aquaticum* Strain 22A, isolated from *Racomitrium japonicum* Moss. Genome Announcements. 3:e00266–15.
2. Almeida, D.M., F.D. Andreote, A.A.C. Neves, et al. 2013. Draft genome sequence of *Methylobacterium mesophilicum* strain SR1.6/6, ssolated from *Citrus sinensis*. Genome Announcements. 1:e00356–13.
3. Anda, M., S. Ikeda, S. Eda, T. Okubo, S. Sato, S. Tabata, H. Mitsui, and K. Minamisawa. 2011. Isolation and genetic characterization of *Aurantimonas* and *Methylobacterium* strains from stems of hypernodulated soybeans. Microbes Environ. 26:172–180.
4. Ardley, J., R. Tian, J. Howieson, et al. 2014. Genome sequence of the dark pink pigmented *Listia bainesii* microsymbiont *Methylobacterium* sp. WSM2598. Stand. Genomic Sci. 9:5.
5. Eevers, N., J.D.V. Hamme, E.M. Bottos, N. Weyens, and J. Vangronsveld. 2015. Draft genome sequence of *Methylobacterium radiotolerans*, a DDE-degrading and plant growth-promoting strain ssolated from *Cucurbita pepo*. Genome Announcements. 3:e004881–5.
6. Elbeltagy, A., K. Nishioka, H. Suzuki, T. Sato, Y. Sato, H. Morisaki, H. Mitsui, and K. Minamisawa. 2000. Isolation and characterization of endophytic bacteria from wild and traditionally cultivated rice varieties. Soil Sci. Plant Nutr. 46:617–629.
7. Finan, T.M., B. Kunkel, G.F. De Vos, E.R. Signer. 1986. Second symbiotic megaplasmid in *Rhizobium meliloti* carrying exopolysaccharide and thiamine synthesis genes. J. Bacteriol. 167:66–72.
8. Fujinami, S., K.T. Yano, T. Onodera, K. Satoh, T. Shimizu, Y. Wakabayashi, I. Narumi, A. Nakamura, and M. Ito. 2015. Draft Genome Sequence of *Methylobacterium* sp. ME121, isolated from soil as a mixed single colony with *Kaistia* sp. 32K. Genome Announcements. 3: e01005-15.
9. Gan, H.M., T.H. Chew, A.O. Hudson, and M.A. Savk. 2012. Genome sequence of *Methylobacterium* sp. strain GXF4, a xylem associated bacterium isolated from *Vitis vinifera* L. grapevine. J. Bacteriol. 194: 5157–5158.
10. Kaneko, T., Y. Nakamura, S. Sato, et al. 2000. Complete genome structure of the nitrogen-fixing symbiotic bacterium *Mesorhizobium loti*. DNA Res. 7:331–338.

11. Koskimäki, J.J., A.M. Pirttilä, E.L. Ihantola, O. Halonen, and A.C. Frank. 2015. The intracellular scots pine shoot symbiont *Methylobacterium extorquens* DSM13060 aggregates around the host nucleus and encodes eukaryote-like proteins. mBio. 6: e00039–15.
12. Kwak, M.J., H. Jeong, M. Madhaiyan, Y. Lee, T.M. Sa, T.K. Oh, and J.F. Kim. 2014. Genome information of *Methylobacterium oryzae*, a plant-probiotic methylotroph in the phyllosphere. PLOS One. 9:e106704.
13. Madhaiyan, M., K.L. Chan, and L. Ji. 2014. Draft genome sequence of *Methylobacterium* sp. strain L2-4, a leaf associated endophytic N-fixing bacterium isolated from *Jatropha curcas* L. Genome Announcements. 2:e01306–14.
14. Marx, C.J., F. Bringel, L. Chistoserdova, et al. 2012. Complete genome sequences of six strains of the genus *Methylobacterium*. J. Bacteriol. 194:4746–4748.
15. Meyer, F., D. Paarman, M.D. Souza, et al. 2008. The metagenomics RAST server – a public resource for the automatic phylogenetic and functional analysis of metagenomes. BMC Bioinf. 9:386.
16. Minami, T., Y. Ohtsubo, M. Anda, Y. Nagata, M. Tsuda, H. Mitsui, M. Sugawara, and K. Minamisawa. 2016. Complete genome sequence of *Methylobacterium* sp. strain AMS5, an isolate from a soybean stem. Genome Announcements. 4:e00144–16.
17. Ohtsubo, Y., W.I. Ohtsubo, Y. Nagata, and M. Tsuda. 2008. GenomeMatcher: A graphical user interface for DNA sequence comparison. BMC Bioinf. 9:376.
18. Okubo, T., S. Ikeda, K. Sasaki, K. Ohshima, M. Hattori, T. Sato, and K. Minamisawa. 2014. Phylogeny and functions of bacterial communities associated with field-grown rice shoots. Microbes Environ. 29:329–332.
19. Peel, D., and J.R. Quayle. 1961. Microbial growth on C1 compounds. I. Isolation and characterization of *Pseudomonas* AM1. Biochem. J. 81:465.
20. Vuilleumier, S., L. Chistoserdova, M.C. Lee, et al. 2009. *Methylobacterium* genome sequences: a reference blueprint to investigate microbial metabolism of C1 compounds from natural and industrial sources. PLOS one. 4:e5584.
21. Wilson, K.J., A. Sessitsch, J.C. Corbo, K.E Giller, A.D.L. Akkermans, and R.A. Jefferson. 1995. β-Glucuronidase (GUS) transposons for ecological and genetic studies of rhizobia and other Gram-negative bacteria. Microbiology 141:1691–1705.
